# Burden of 46 key pathogens among children in G20 countries, 2023: A systematic analysis and regional comparison

**DOI:** 10.1371/journal.pntd.0014723

**Published:** 2026-09-17

**Authors:** Yonghan Luo, Ying Zhu, Haifeng Jin, Penghao Cui, Ruonan Li, Qingping Tang, Yin Li, Yanchun Wang

**Affiliations:** 1 Second Department of Infectious Disease, Children’s Hospital Affiliated to Kunming Medical University (Kunming Children’s Hospital), Kunming, Yunnan, China; 2 Faculty of Life Science and Technology, Kunming University of Science and Technology, Kunming, Yunnan, China; Yale University School of Medicine, UNITED STATES OF AMERICA

## Abstract

**Background:**

Infectious diseases remain a major public health threat to child survival and health, with particularly substantial burdens attributable to lower respiratory infections, diarrheal diseases, and CNS infections. Although the Global Burden of Disease (GBD) studies have previously evaluated specific pathogens or individual clinical syndromes, a descriptive assessment of the burden attributable to major pathogens across multiple infectious syndromes in children within G20 countries remains limited. Using the most recent GBD 2023 dataset, this study aimed to systematically evaluate the burden and temporal trends of 46 key pathogens among children aged 0–14 years in G20 countries from 1990 to 2023.

**Methods:**

Data were obtained from the Global Burden of Disease Study 2023 (GBD 2023). We extracted estimates of deaths and disability-adjusted life years (DALYs) attributable to 46 pathogens among children aged 0–14 years in G20 countries between 1990 and 2023, and calculated corresponding death and DALY rates. Temporal trends were assessed using the estimated annual percentage change (EAPC). Regional disparities were examined through stratified analyses based on the Socio-demographic Index (SDI). The principal infectious syndromes analyzed included lower respiratory infections, diarrheal diseases, and meningitis.

**Results:**

In 2023, *Streptococcus pneumoniae* was the leading pathogen contributing to death and DALY loss among children in G20 countries, accounting for approximately 66,400 deaths (95% UI: 56,400–77,000) and 5.90 million DALYs (95% UI: 5.01–6.85 million). It also represented the predominant pathogen responsible for meningitis-related burden. *Klebsiella pneumoniae* ranked second, causing approximately 21,500 deaths (95% UI: 17,900–24,700), followed closely by Adenovirus (approximately 16,700 deaths; 95% UI: 9,300–26,200) and Rotavirus (approximately 16,100 deaths; 95% UI: 11,100–22,900). In terms of temporal trends, death from *Haemophilus influenzae* meningitis exhibited the most pronounced decline, with an average annual reduction of 9.06% (95% CI: −9.98 to −8.14). Regional analyses indicated that India, South Africa, and Indonesia bore the greatest burden within the G20. Age-stratified analyses revealed that infants younger than one year experienced substantially higher disease burdens than other pediatric age groups. Correlation analyses further demonstrated a significant inverse relationship between SDI and pathogen-attributable death rates (r = −0.8456, p < 0.001).

**Conclusions:**

Although the overall burden of pediatric infectious diseases in G20 countries has declined markedly since 1990, substantial burdens attributable to pathogens such as *Streptococcus pneumoniae*, rotavirus, and *Klebsiella pneumoniae* persist in lower-SDI member states. Moving forward, priority should be given to increasing coverage of pneumococcal conjugate vaccines and rotavirus vaccines in resource-limited G20 settings, strengthening pathogen surveillance, and improving access to timely diagnosis and treatment for severe infections in order to further reduce health inequalities.

## Author summary

Infectious diseases remain a major threat to children‘s health worldwide, including within the G20 countries. Using data from the Global Burden of Disease Study 2023, we analyzed deaths and disabilities caused by 46 different pathogens (bacteria and viruses) in children aged 0–14 years across G20 member states. We found that in 2023, *Streptococcus pneumoniae* (the bacterium that causes pneumonia and meningitis) was the leading killer of children in the G20, followed by *Klebsiella pneumoniae* and Adenovirus, with Rotavirus also contributing substantially to diarrheal deaths. The burden was highest in infants under one year old and in G20 countries with lower socio-demographic index, particularly India, South Africa, and Indonesia. Encouragingly, the overall disease burden has declined substantially since 1990, with the largest drop seen in *Haemophilus influenzae* meningitis. However, large gaps remain across G20 members. Our findings highlight the urgent need to expand pneumococcal and rotavirus vaccines in lower-resource G20 settings, improve access to clean water and sanitation, and strengthen healthcare systems to reduce preventable child deaths.

## 1 Introduction

Infectious diseases remain one of the central public health threats to child health and survival, with the burden of death and disability remaining persistently high among children under five years of age [1]. Pediatric infections not only lead to acute death but may also cause long-term health impairments through recurrent episodes and severe disease processes, including growth retardation and compromised immune function, thereby substantially reducing healthy life expectancy and overall quality of life. From an etiological perspective, viruses and bacteria constitute the predominant pathogenic groups responsible for pediatric infectious diseases. Viruses play a prominent role in community transmission and seasonal outbreaks and are frequently implicated in acute respiratory and gastrointestinal infections, whereas bacterial pathogens are more commonly associated with severe pneumonia, sepsis, invasive infections, and fatal outcomes [2].

With respect to anatomical sites of infection, the most common foci in children are the respiratory and gastrointestinal tracts. Lower respiratory infections (LRIs) and infectious diarrhea represent two major syndromic contributors to the pediatric disease burden [3,4]. In contrast, although central nervous system (CNS) infections occur less frequently overall, they are associated with substantially higher case-fatality rates and a greater risk of long-term neurological sequelae, and therefore represent some of the most severe manifestations of pediatric infectious disease [5]. Consequently, a systematic and comprehensive characterization of the differential disease burden attributable to diverse pathogens across distinct infection syndromes in children is essential for optimizing vaccination strategies, guiding antimicrobial therapy, and informing the allocation of healthcare resources.

The Global Burden of Disease (GBD) study [6,7] establishes a standardized analytical framework based on multicenter data, generating key metrics such as death, disability-adjusted life years (DALYs), and rates, thereby providing a unified platform for comparisons across countries, regions, and time periods.

In recent years, several studies based on GBD data have described the burden of specific pathogens or individual infectious syndromes in children. For instance, Wang et al. [8] evaluated the global burden attributable to *Streptococcus pneumoniae* among individuals younger than 20 years from 1990 to 2021 and conducted predictive analyses. Yi et al. [9] characterized the burden and inequalities of LRIs among children and adolescents from global to subnational levels, enabling comparisons across multiple geographic scales. Regarding diarrheal diseases, Tian et al. [10] systematically analyzed long-term trends, disparities, and pathogen spectrum changes in infectious diarrhea among children under five years of age between 1990 and 2021, while Wang et al. [11] further examined childhood diarrhea attributable to pathogenic microorganisms with age-stratified analyses and evaluation of risk factors. In the domain of CNS infections, Yang et al. [12] reported global, regional, and national trends in meningitis among children aged 0–14 years. Li et al. further analyzed the burden and etiological composition of pediatric meningitis from 1990 to 2021, highlighting the important contributions of *Streptococcus pneumoniae*, *Neisseria meningitidis*, and *Klebsiella pneumoniae* to death [5]. In addition, Luo et al. [13] conducted a systematic assessment and projection of the burden of infectious encephalitis among children in Asia, emphasizing that regions with low Socio-demographic Index (SDI) continue to shoulder disproportionately high disease burdens. While these investigations provide valuable insights into specific disease spectra or individual pathogens, a notable gap remains: the absence of an integrated assessment of pathogen-specific burdens across multiple major infectious syndromes in the pediatric population.

Compared with the pediatric field, integrated pathogen-level assessments across multiple diseases have already been explored in adult populations, particularly among the elderly. Chen et al. [14], based on GBD 2021 data, systematically evaluated the global and regional death and DALY burdens attributable to 34 major bacterial and viral pathogens in older adults. However, a comparable comprehensive pathogen-level assessment focusing specifically on children remains lacking. Furthermore, most previous pediatric burden analyses relied on data up to 2021. The COVID-19 pandemic, together with related non-pharmaceutical interventions and shifts in pathogen transmission niches, may have substantially altered the epidemiological patterns of respiratory and enteric pathogens as well as the distribution of severe infections. In this context, GBD 2023 data [15] provide updated evidence closer to the post-pandemic period and may offer a more contemporary basis for informing forward-looking strategies for pediatric infectious disease prevention and control.

G20 member states encompass a wide spectrum of socioeconomic development, ranging from high-income countries to emerging economies, making this grouping particularly valuable for examining the relationship between economic development and pediatric infectious disease burden. In light of these considerations, the present study employs standardized estimates from the GBD 2023 framework to examine 46 key pathogens among children aged 0–14 years in G20 countries. We comprehensively assess their associated death and DALY burdens across major infectious syndromes and compare patterns across GBD regions and SDI strata. We tested the following hypothesis: the burden of pediatric infectious diseases within G20 countries remains disproportionately concentrated in lower-SDI member states, with Streptococcus pneumoniae and rotavirus being the leading pathogens; and despite overall declines since 1990, significant disparities persist across geographic regions and age groups. Through this approach, we aim to generate G20-specific evidence with practical relevance for the surveillance and control of pediatric infectious diseases moving forward.

## 2 Methods

### 2.1 Ethics statement

As this research involved a secondary analysis of publicly available data, institutional review board approval and informed consent were not required. All methods were carried out in accordance with relevant guidelines and regulations or Declaration of Helsinki.

### 2.2 Data source

This is a GBD-based ecological analysis. This study was conducted using data from the Global Burden of Disease Study 2023 (GBD 2023). Relevant data were obtained from the Global Health Data Exchange (GHDx; https://vizhub.healthdata.org/gbd-results/). Access to the GBD 2023 results tool requires free registration; no payment or subscription is required. GBD 2023 provides comprehensive and systematic estimates of disease burden at global, regional, and national levels. The present analysis focused on the burden attributable to 46 major bacterial and viral pathogens among children aged 0–14 years in G20 countries from 1990 to 2023, encompassing death and DALYs. In addition, a cross-sectional analysis of 2023 data was performed to evaluate contemporary patterns and regional disparities in disease burden across G20 member states. As this investigation was based exclusively on publicly accessible secondary data and did not involve identifiable individual information, institutional ethics approval and informed consent were not required.

### 2.3 Data extraction and variable definitions

Death and DALY data attributable to 46 pathogens among children were extracted from the GBD database at global, regional, and national levels for G20 countries. The G20 member states included in this analysis were: Argentina, Australia, Brazil, Canada, China, France, Germany, India, Indonesia, Italy, Japan, Mexico, Russia, Saudi Arabia, South Africa, South Korea, Turkey, the United Kingdom, the United States, and the European Union (as a bloc). The analytical framework encompassed estimates for G20 countries as a whole as well as individual member states. The disease categories primarily included pathogen-attributable burdens of LRIs, diarrheal diseases, and meningitis. Sex was specified as “Both,” indicating combined analyses of males and females. Children were defined as individuals aged 0–14 years and were further stratified into five age groups: < 1 year, 1–2 years, 2–4 years, 5–9 years, and 10–14 years. The study period spanned from 1990 to 2023 to facilitate temporal trend analyses and annual comparisons. Primary outcome measures included the number of deaths, total DALYs, death rates, and DALY rates. All rates were expressed per 100,000 population and reported with 95% uncertainty intervals (95% UI).

### 2.4 Statistical analysis

All statistical analyses were conducted using R software (version 4.4.2). Based on data extracted from the GBD database, the numbers of deaths, DALYs, and corresponding rates attributable to the 46 pathogens were calculated and compared across G20 countries and regions.

To quantify temporal trends in pathogen-attributable death and DALY rates among children aged 0–14 years in G20 countries from 1990 to 2023, we calculated the Estimated Annual Percentage Change (EAPC). Using year as the independent variable, we applied a logarithmic transformation to rates. The geometric mean for each year was taken, and this sequence of geometric means was treated as the dependent variable to fit a linear regression model. The EAPC was calculated using the formula 100 × (exp(β) − 1), where β represents the regression coefficient. The 95% confidence interval (CI) for the EAPC was derived from the linear regression model. A positive EAPC with its 95% CI entirely above zero indicates an increasing trend, while a negative EAPC with its 95% CI entirely below zero indicates a decreasing trend. All burden estimates (deaths, DALYs, death rates, and DALY rates) are presented with 95% uncertainty intervals (95% UI) as provided by GBD 2023. To evaluate the association between the SDI and disease burden, due to the non-normal distribution of the data, Spearman‘s rank correlation test was performed. A two-sided P value <0.05 was considered statistically significant. Given the exploratory and descriptive nature of this study, which analyzed 46 pathogens across multiple syndromes and time points, we did not adjust the alpha level for multiple comparisons. Readers should interpret the findings with caution regarding the potential risk of type I error.

## 3 Results

### 3.1 Global burden of 46 pathogens in g20 countries in 2023

In 2023, Streptococcus pneumoniae was the leading contributor to the disease burden in G20 countries, accounting for 66,394.314 deaths (95% UI: 56,369.219–76,988.586) and 5,901,914.988 DALYs (95% UI: 5,011,319.774–6,845,714.670), with a death rate of 6.873 per 100,000 (95% UI: 5.836–7.970) and a DALY rate of 610.991 per 100,000 (95% UI: 518.786–708.709). Klebsiella pneumoniae ranked second (21,481.923 deaths; rate: 2.224 per 100,000; 1,917,687.981 DALYs; DALY rate: 198.531 per 100,000), and Adenovirus ranked third (16,659.888 deaths; rate: 1.725 per 100,000; 1,610,937.727 DALYs; DALY rate: 166.774 per 100,000), closely followed by Rotavirus (16,125.003 deaths; rate: 1.669 per 100,000; 1,614,105.070 DALYs; DALY rate: 167.102 per 100,000). In contrast, Clostridium difficile and several other pathogens (e.g., Legionella spp., Morganella spp., Proteus spp., Serratia spp., Citrobacter spp.) were associated with the lowest burden.

When ranked by number of deaths globally, the ten leading pathogens among children aged 0–14 years were: Streptococcus pneumoniae, Klebsiella pneumoniae, Adenovirus, Rotavirus, Pseudomonas aeruginosa, Staphylococcus aureus, Shigella, Enterotoxigenic E. coli (ETEC), Enteropathogenic E. coli (EPEC), and Cryptosporidium. When ranked by DALYs, the top ten pathogens were: Streptococcus pneumoniae, Klebsiella pneumoniae, Rotavirus, Adenovirus, Pseudomonas aeruginosa, Shigella, Staphylococcus aureus, Enterotoxigenic E. coli (ETEC), Enteropathogenic E. coli (EPEC), and Cryptosporidium. Detailed estimates of top ten pathogen-specific deaths and corresponding rates are presented in **Table 1**, while DALYs and their rates are summarized in **Table 2**. Complete estimates for all 46 pathogens are available in **S1** **and** **S2 Tables**.

**Table 1:** Global deaths and death rates for top ten pathogens among children aged 0–14 years, by infectious syndrome, 2023.

Pathogen	All 3 infectious syndromes		Lower respiratory infections		Diarrheal diseases		Meningitis	
	Death cases	Rate per 100,000 (95%UI)	Death cases	Rate per 100,000 (95%UI)	Death cases	Rate per 100,000 (95%UI)	Death cases	Rate per 100,000 (95%UI)
Adenovirus	16659.888 (9316.318 - 26158.864)	1.725 (0.964 - 2.708)			16659.888 (9316.318 - 26158.864)	1.725 (0.964 - 2.708)		
Cryptosporidium	5243.993 (2813.936 - 8846.671)	0.543 (0.291 - 0.916)			5243.993 (2813.936 - 8846.671)	0.543 (0.291 - 0.916)		
Enteropathogenic E coli	5480.856 (2733.926 - 9622.492)	0.567 (0.283 - 0.996)			5480.856 (2733.926 - 9622.492)	0.567 (0.283 - 0.996)		
Enterotoxigenic E coli	7068.311 (4182.515 - 11086.358)	0.732 (0.433 - 1.148)			7068.311 (4182.515 - 11086.358)	0.732 (0.433 - 1.148)		
Klebsiella pneumoniae	21481.923 (17948.480 - 24660.640)	2.224 (1.859 - 2.553)	20930.093 (17508.200 - 23940.873)	2.167 (1.813 - 2.478)			551.830 (440.280 - 719.767)	0.057 (0.046 - 0.075)
Pseudomonas aeruginosa	13010.717 (11023.146 - 15006.662)	1.347 (1.141 - 1.553)	12564.568 (10668.815 - 14425.663)	1.301 (1.104 - 1.493)			446.149 (354.331 - 580.999)	0.046 (0.037 - 0.060)
Rotavirus	16125.003 (11124.277 - 22903.026)	1.669 (1.152 - 2.371)			16125.003 (11124.277 - 22903.026)	1.669 (1.152 - 2.371)		
Staphylococcus aureus	10500.983 (8993.304 - 11898.438)	1.088 (0.931 - 1.232)	10050.714 (8620.312 - 11341.994)	1.041 (0.892 - 1.174)			450.269 (372.992 - 556.444)	0.047 (0.039 - 0.058)
Streptococcus pneumoniae	66394.314 (56369.219 - 76988.586)	6.873 (5.836 - 7.970)	63822.665 (54264.434 - 73576.289)	6.607 (5.618 - 7.617)			2571.649 (2104.785 - 3412.297)	0.266 (0.218 - 0.353)
Shigella	9736.276 (5712.697 - 15428.745)	1.008 (0.591 - 1.597)			9736.276 (5712.697 - 15428.745)	1.008 (0.591 - 1.597)		

Note: All values are modelled estimates with 95% uncertainty intervals (95% UI) as provided by the Global Burden of Disease Study 2023 (GBD 2023).

**Table 2:** Global disability-adjusted life years (DALYs) and DALY rates for top ten pathogens among children aged 0–14 years, by infectious syndrome, 2023.

Pathogen	All 3 infectious syndromes		Lower respiratory infections		Diarrheal diseases		Meningitis	
	DALYs cases (95%UI)	Rate per 100,000 (95%UI)	DALYs cases (95%UI)	Rate per 100,000 (95%UI)	DALYs cases (95%UI)	Rate per 100,000 (95%UI)	DALYs cases (95%UI)	Rate per 100,000 (95%UI)
Adenovirus	1610937.727 (977911.674 - 2480813.727)	166.774 (101.239 - 256.828)			1610937.727 (977911.674 - 2480813.727)	166.774 (101.239 - 256.828)		
Cryptosporidium	492729.502 (267120.397 - 819288.296)	51.010 (27.654 - 84.817)			492729.502 (267120.397 - 819288.296)	51.010 (27.654 - 84.817)		
Enteropathogenic E coli	516592.333 (263322.019 - 890236.499)	53.481 (27.261 - 92.162)			516592.333 (263322.019 - 890236.499)	53.481 (27.261 - 92.162)		
Enterotoxigenic E coli	704750.874 (436825.062 - 1089053.840)	72.960 (45.223 - 112.745)			704750.874 (436825.062 - 1089053.840)	72.960 (45.223 - 112.745)		
Klebsiella pneumoniae	1917687.981 (1600781.719 - 2202024.594)	198.531 (165.722 - 227.962)	1868273.365 (1561100.746 - 2137476.052)	193.415 (161.614 - 221.284)			49414.616 (39680.973 - 64508.542)	5.116 (4.108 - 6.678)
Pseudomonas aeruginosa	1160415.500 (982146.395 - 1339291.581)	120.133 (101.681 - 138.651)	1120336.794 (951393.589 - 1286881.461)	115.984 (98.494 - 133.225)			40078.706 (31752.806 - 52410.120)	4.149 (3.287 - 5.426)
Rotavirus	1614105.070 (1124262.092 - 2248233.149)	167.102 (116.390 - 232.750)			1614105.070 (1124262.092 - 2248233.149)	167.102 (116.390 - 232.750)		
Staphylococcus aureus	932719.870 (798757.060 - 1057063.416)	96.561 (82.692 - 109.434)	892646.865 (765439.141 - 1007243.871)	92.412 (79.243 - 104.276)			40073.005 (33317.919 - 49819.545)	4.149 (3.449 - 5.158)
Streptococcus pneumoniae	5901914.988 (5011319.774 - 6845714.670)	610.991 (518.786 - 708.709)	5674640.821 (4824710.057 - 6544160.873)	587.472 (499.482 - 677.490)			227274.167 (186609.717 - 301553.797)	23.529 (19.319 - 31.219)
Shigella	986660.385 (603220.226 - 1537554.390)	102.145 (62.449 - 159.177)			986660.385 (603220.226 - 1537554.390)	102.145 (62.449 - 159.177)		

Note: All values are modelled estimates with 95% uncertainty intervals (95% UI) as provided by the Global Burden of Disease Study 2023 (GBD 2023).

### 3.2 Temporal trends in the global burden of 46 pathogens (1990–2023)

**Fig 1:**
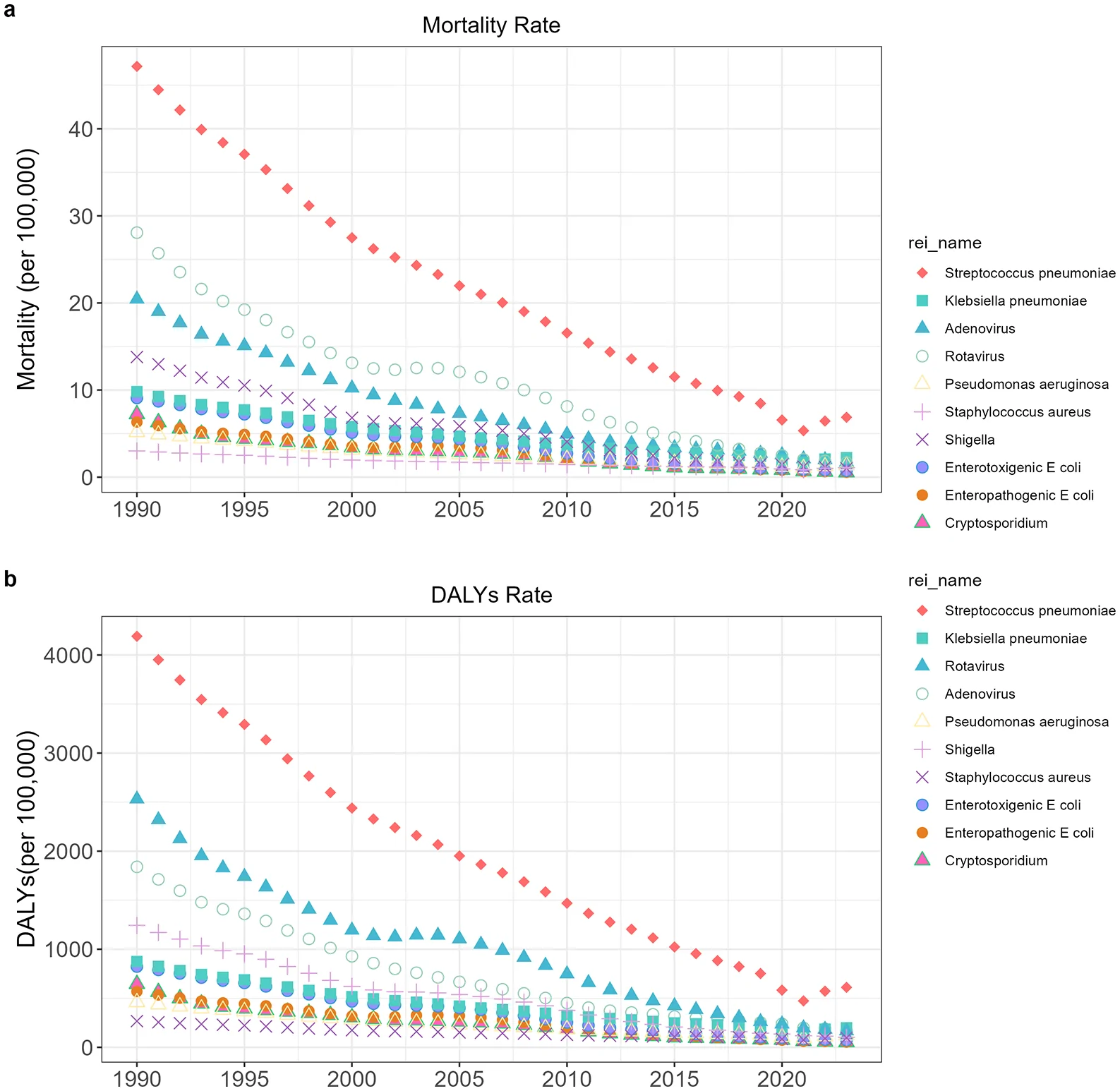
Temporal trends in pathogen-attributable death and DALY rates among children in G20 countries, 1990–2023. (a) Trends in death rates (per 100,000 population) for the top ten pathogens in 2023 among children aged 0–14 years in G20 countries. Lines represent individual pathogens.(b) Corresponding trends in DALY rates (per 100,000 population) for the same pathogens.

From 1990 to 2023, the overall burden demonstrated a marked declining trend across G20 countries **(**Fig 1a**–**1b).

The most pronounced reduction was observed for H. influenzae meningitis (death rate: from 1.388 to 0.063 per 100,000; EAPC: −9.06; DALY rate: from 122.942 to 5.742 per 100,000; EAPC: −9.01), followed by Campylobacter (death rate: from 7.928 to 0.515 per 100,000; EAPC: −7.76; DALY rate: from 720.708 to 53.558 per 100,000; EAPC: −7.47) and non-polio enteroviruses meningitis (death rate: from 1.153 to 0.088 per 100,000; EAPC: −7.72; DALY rate: from 107.732 to 9.487 per 100,000; EAPC: −7.35). In contrast, Legionella spp. showed a slight increasing trend (death rate: from 0.015 to 0.028 per 100,000; EAPC: 2.00; DALY rate: from 1.284 to 2.377 per 100,000; EAPC: 1.95), as did Clostridium difficile (death rate: from 0.009 to 0.012 per 100,000; EAPC: 1.22; DALY rate: from 0.752 to 1.006 per 100,000; EAPC: 1.22). Detailed EAPC estimates for each pathogen are presented in **S3 Table** **A-B**.

### 3.3 National disparities in the burden attributable to 46 pathogens in G20 countries

**Fig 2:**
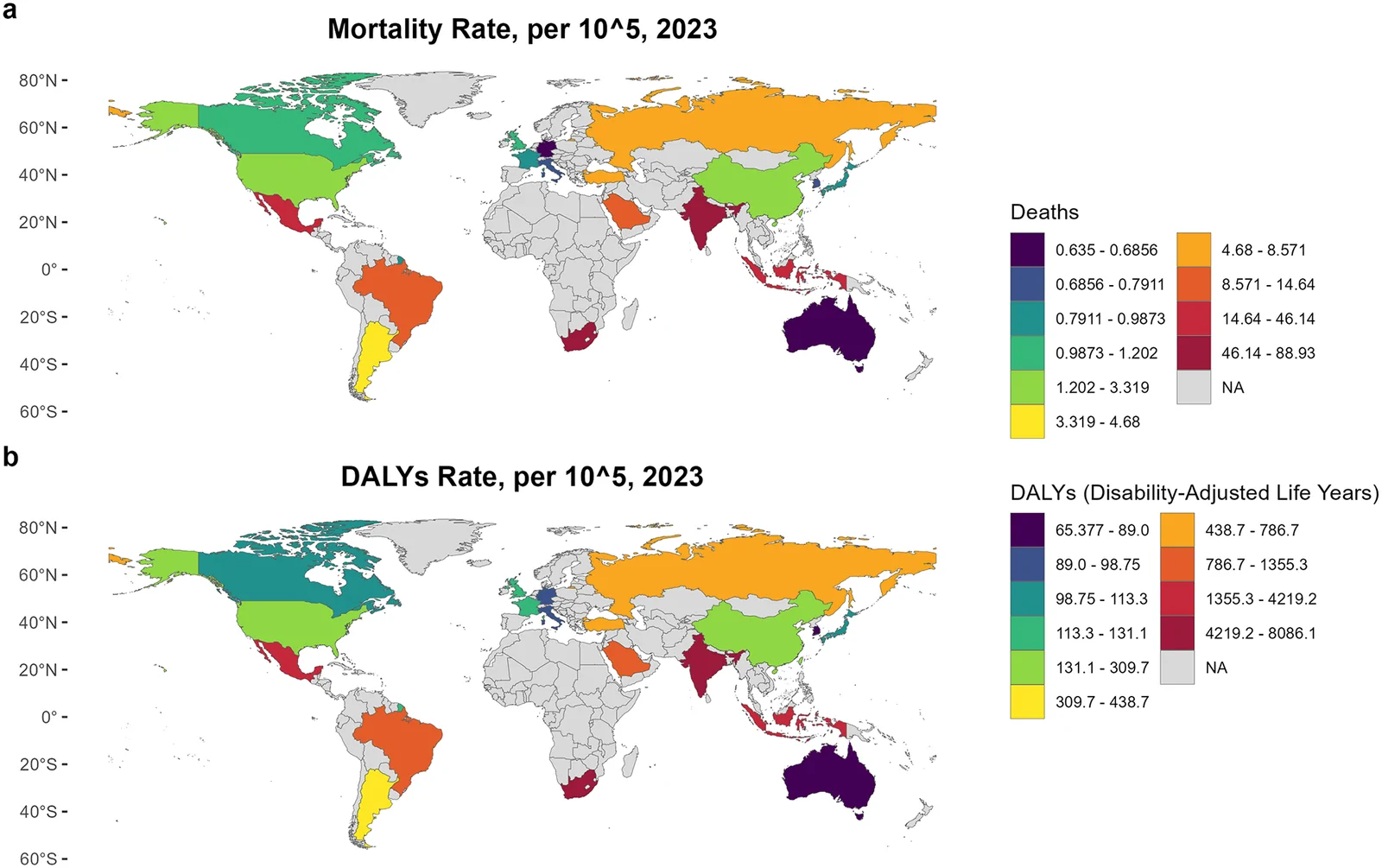
Geographic distribution of pathogen-attributable death and DALY rates among children in G20 countries in 2023. (a) Country-level death rates (per 100,000 population) attributable to major pathogens among children aged 0–14 years. Color intensity increases with rate.(b) Country-level DALY rates (per 100,000 population) for the same year. Color intensity increases with rate. The base map layer was obtained from Natural Earth Data (https://www.naturalearthdata.com/) and created using R software.

**Fig 3:**
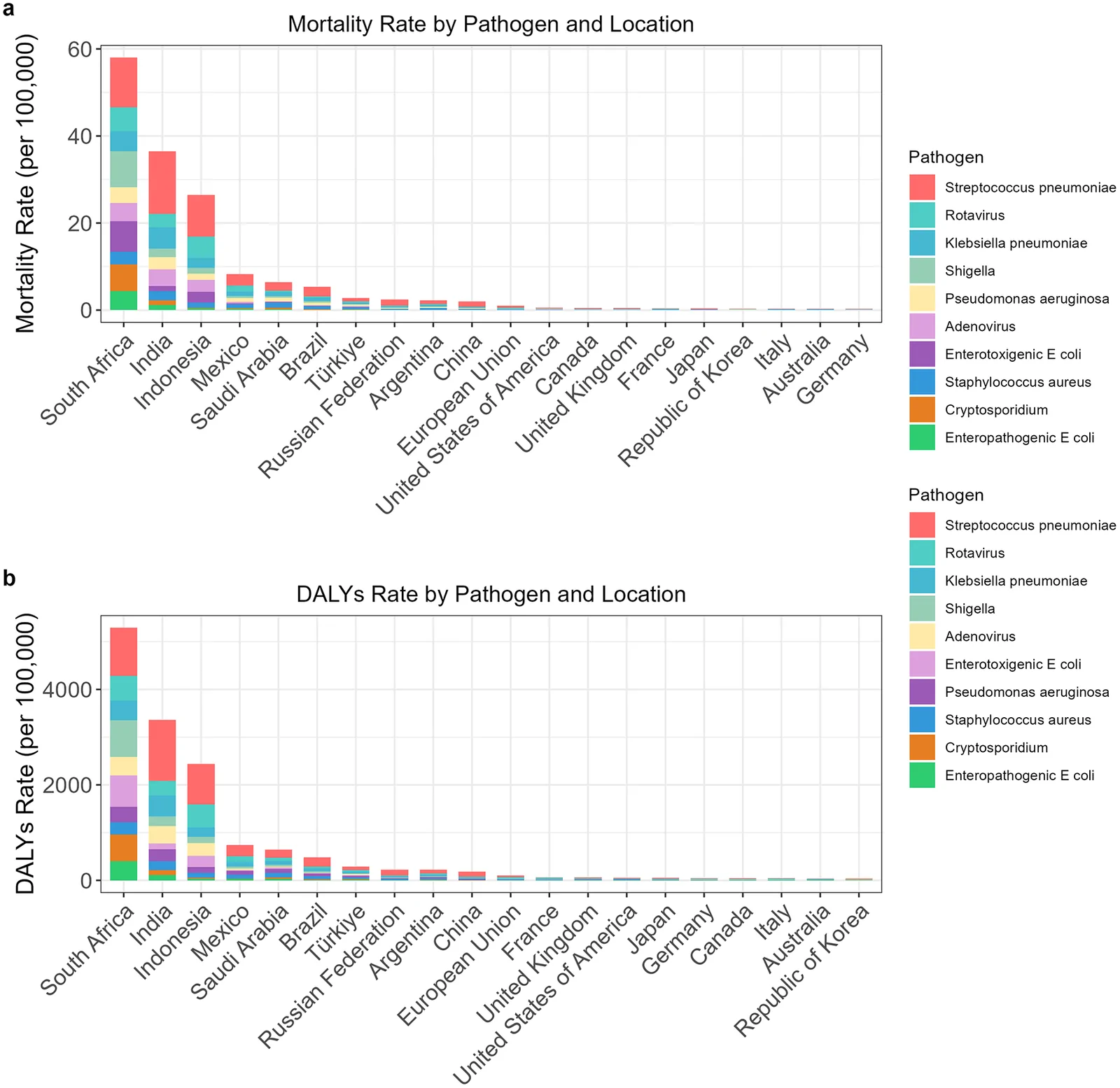
Regional distribution of pathogen-attributable death and DALY rates among children in G20 countries in 2023. (a) Death rates (per 100,000 population) across G20 member states and Socio-demographic Index (SDI) groups.(b) Corresponding DALY rates (per 100,000 population) across the same regions and SDI categories.

In 2023, India reported the highest burden, with 207,890 deaths (rate: 57.329 per 100,000) and 19.13 million DALYs (rate: 5,276.606 per 100,000), followed by South Africa (14,312 deaths; rate: 88.934 per 100,000; 1.30 million DALYs; rate: 8,086.118 per 100,000). By contrast, the Republic of Korea (39 deaths; rate: 0.691 per 100,000) and Australia (32 deaths; rate: 0.663 per 100,000) recorded the lowest burdens among G20 members, with rates below 1 per 100,000. **(**Fig 2a**–2b) (**Fig 3a**–**3b**).**

### 3.4 Age-specific differences in the burden attributable to 46 pathogens in G20 countries

**Fig 4:**
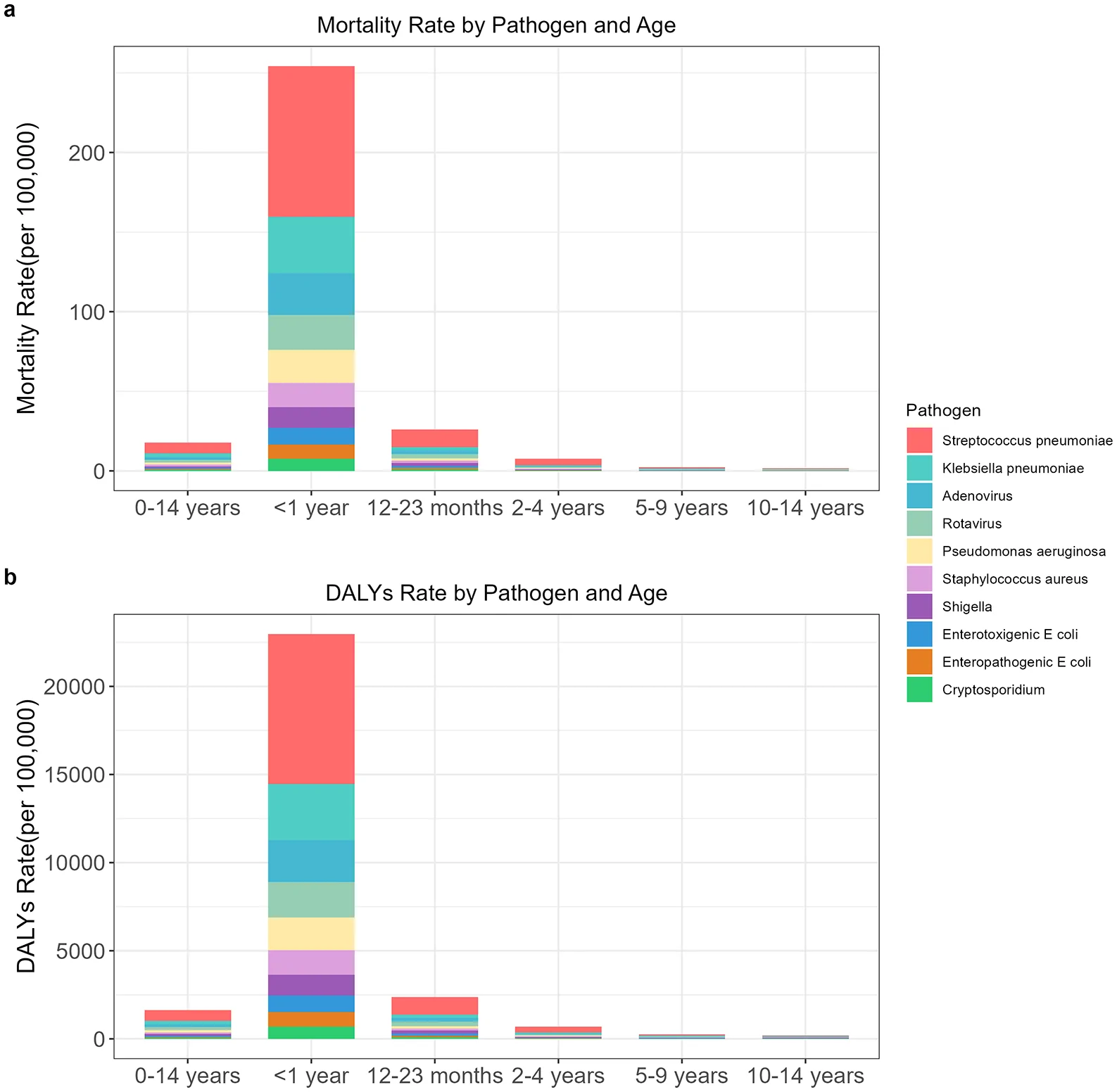
Age-stratified death and DALY rates attributable to major pathogens among children in G20 countries in 2023. (a) Death rates (per 100,000 population) by age group (<1 year, 12–23 months, 2–4 years, 5–9 years, 10–14 years, and overall 0–14 years). Stacked bars represent cumulative rates, with each color representing a specific pathogen.(b) Corresponding DALY rates (per 100,000 population) across the same age groups.

S. pneumoniae was the predominant pathogen across all age groups, with the highest burden in infants aged <1 year (48,911.120 deaths; rate: 94.766 per 100,000; 4,390,299.471 DALYs; DALY rate: 8,506.573 per 100,000). Burden decreased sharply with age: 2–4 years (6,908.431 deaths; rate: 5.884 per 100,000; 608,222.656 DALYs; DALY rate: 517.979 per 100,000), 5–9 years (2,538.891 deaths; rate: 0.742 per 100,000; 215,249.646 DALYs; DALY rate: 62.921 per 100,000), and 10–14 years (2,072.377 deaths; rate: 0.604 per 100,000; 165,823.638 DALYs; DALY rate: 48.355 per 100,000). Beginning in the 5–9-year group, Shigella contributed more prominently, though overall death was substantially lower than in children under 5 years **(**Fig 4a**–**4b).

### 3.5 Pathogen-specific burden across disease

**Fig 5:**
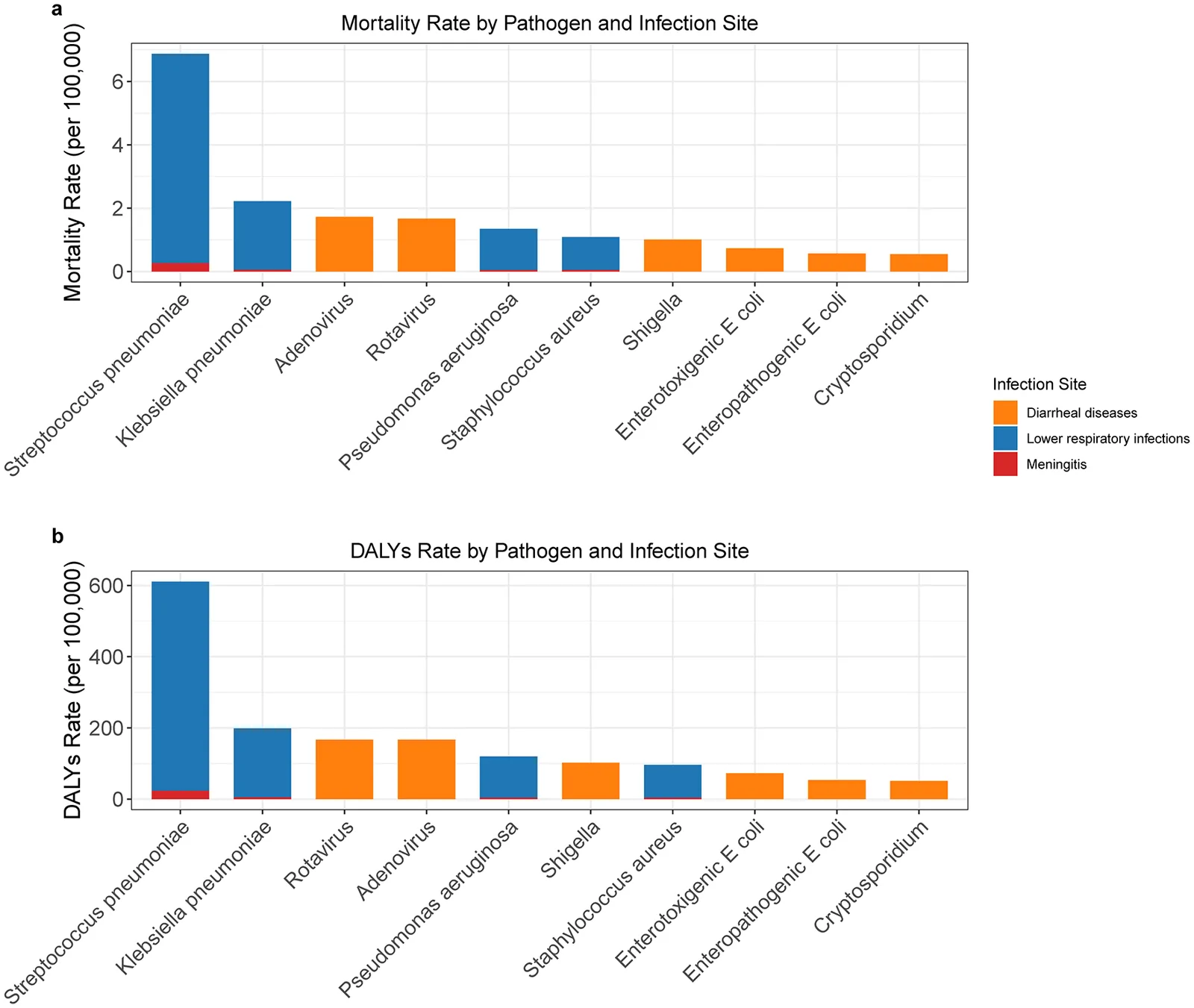
Death and DALY rates attributable to major pathogens stratified by infection site among children in G20 countries in 2023. (a) Death rates (per 100,000 population) by infection site (lower respiratory infections, diarrheal diseases, and meningitis). (b) Corresponding DALY rates (per 100,000 population) by infection site.

In 2023, pathogen-attributable burden varied substantially across LRIs, diarrheal diseases, and meningitis **(**Fig 5a**–**5b):

LRIs: S. pneumoniae was the leading cause (63,822.665 deaths; rate: 6.607 per 100,000; 5,674,640.821 DALYs; DALY rate: 587.472 per 100,000), while Legionella spp. had the lowest burden (265.859 deaths; rate: 0.028 per 100,000; 22,960.358 DALYs; DALY rate: 2.377 per 100,000).

Diarrheal diseases: Rotavirus ranked first (16,125.003 deaths; rate: 1.669 per 100,000; 1,614,105.070 DALYs; DALY rate: 167.102 per 100,000), with C. difficile the lowest (115.709 deaths; rate: 0.012 per 100,000; 9,721.637 DALYs; DALY rate: 1.006 per 100,000).

Meningitis: S. pneumoniae again ranked first (2,571.649 deaths; rate: 0.266 per 100,000; 227,274.167 DALYs; DALY rate: 23.529 per 100,000), while E. coli had the lowest burden among meningitis pathogens (348.835 deaths; rate: 0.036 per 100,000; 31,241.344 DALYs; DALY rate: 3.234 per 100,000).

### 3.6 Trends and correlations in pathogen burden across socio-demographic index (SDI) levels

**Fig 6:**
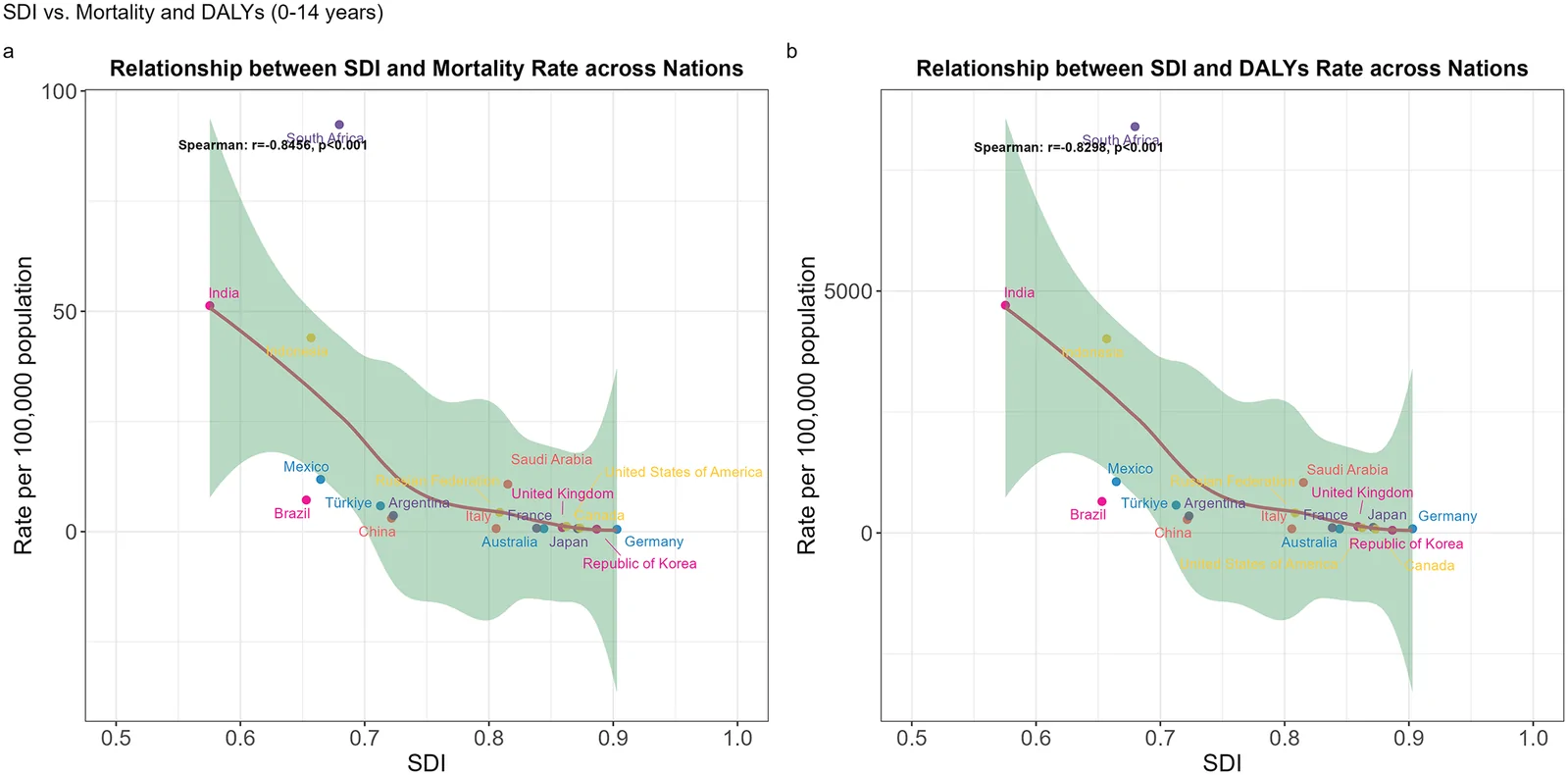
Relationship between Socio-demographic Index (SDI) and pathogen-attributable burden among children in G20 countries, 2023. Scatter plots showing the correlation between SDI and (a) death rates and (b) DALY rates. Each point represents a G20 member state. Spearman’s correlation coefficients (*r*) and -values are shown in each panel.

At the G20 country level, the burden attributable to the 46 pathogens among children in 2023 exhibited a pronounced SDI gradient. Fig 6a**–**6b demonstrated a highly consistent inverse association between SDI and pathogen-attributable burden across G20 countries. Spearman’s correlation coefficients were r = −0.8456 (p < 0.001) for SDI versus death rate and r= −0.8298 (p < 0.001) for SDI versus DALY rate, indicating that lower SDI was strongly associated with higher childhood death and DALY rates. This relationship remained robust across G20 member states. These findings indicate that childhood pathogen-attributable death and DALY burdens in G20 countries are geographically concentrated in lower-SDI member states and are strongly and consistently negatively correlated with SDI.

## 4 Discussion

Using the standardized estimation framework of GBD 2023, this study systematically assessed the attributable burden of 46 key pathogens across major infection sites among children aged 0–14 years in G20 countries. The principal indicators included numbers of deaths, DALYs, and their corresponding rates, with additional stratified comparisons across G20 member states and SDI levels. Overall, our findings indicate that the burden of pediatric infectious diseases within the G20 remains predominantly concentrated in pathogens associated with LRIs and diarrheal diseases, with a pronounced clustering in lower-SDI G20 members. Furthermore, the contributions of specific pathogens vary substantially across different infection syndromes. These observations are consistent with previous GBD 2021–based analyses of pediatric LRIs and diarrheal diseases that documented persistent global inequalities in disease burden [9,10], and they also align with findings from studies of pediatric infectious encephalitis in Asia demonstrating an inverse relationship with SDI [13]. Collectively, these results underscore that future interventions within the G20 should prioritize high-burden pathogens while simultaneously improving vaccine accessibility, early detection, and standardized treatment in lower-SDI member states in order to maximize reductions in preventable death and DALYs.

Geographically, the burden was disproportionately concentrated in lower-SDI G20 members, particularly India, South Africa, and Indonesia. This spatial clustering **(**Fig 2a**–**2b) reflects the convergence of poverty, weak health systems, and limited vaccine access in these regions, creating a “childhood infectious disease belt” within the G20 where multiple pathogens co-circulate at high intensity. Large-scale clinical studies from China have further highlighted the severe consequences of pneumococcal meningitis in children, reporting high rates of neurological complications and long-term sequelae [16,17]. At the G20 level, etiological analyses also identify *S. pneumoniae* as a major contributor to meningitis-related death [5].

From a geographical perspective, we observed that the burden attributable to pneumococcus closely corresponds with disparities in pneumococcal conjugate vaccine (PCV) coverage. Evidence from Uganda (PCV10) [18], Cameroon (PCV13) [19], and Japan (PCV7/PCV13) [20] has demonstrated that PCV introduction is associated with marked declines in pediatric pneumococcal meningitis and overall disease burden. When interpreted alongside our observations of persistent high burden in lower-SDI G20 members—particularly India, Indonesia, and South Africa—these findings collectively highlight PCV scale-up as a critical public health priority within the G20. Future strategies should prioritize increasing PCV accessibility and full-dose coverage in G20 members with low vaccination uptake, while strengthening serotype surveillance to inform next-generation vaccine policies. Our analysis confirms that rotavirus remains the main leading cause of pediatric infectious disease burden in G20 countries, with burden concentrated in lower-SDI member states where vaccine coverage remains suboptimal, with its burden map **(**Fig 2a**–**2b**)** closely mirroring that of pneumococcus—both concentrated in South Asia and sub-Saharan African G20 members (India and South Africa). This overlap suggests shared underlying determinants (poverty, poor sanitation, weak primary care) and supports integrated intervention packages rather than vertical disease programs. Despite the availability of oral rehydration salts (ORS) and rotavirus vaccines, the persistence of substantial death suggests that effective interventions remain insufficiently accessible in high-risk populations. Vaccine impact studies from Mozambique [21], South America [22]., and global systematic reviews [23] have demonstrated that rotavirus vaccination significantly reduces diarrhea-related hospitalizations and death. In the Chinese context, rotavirus is also identified as a major pathogen responsible for diarrhea among children under five [24]. Therefore, in high-burden G20 countries and lower-SDI member states, a combined strategy integrating improved ORS access with expanded rotavirus vaccination is key to reducing preventable death.

With respect to infection syndromes, our findings reaffirm that LRIs contribute the greatest overall burden, diarrheal diseases remain highly significant, and CNS infections, although less frequent, are disproportionately severe. The stacked bar charts **(**Fig 5a**–**5b) reveal that lower respiratory infections account for the largest share of both death and DALYs across pathogens, while meningitis—despite smaller absolute numbers—shows the highest case-fatality ratio per episode, indicating its outsized severity. Infants and young children are particularly vulnerable to severe pneumonia and respiratory failure because of their narrow airways, limited ability to clear respiratory secretions, and immature immune systems. The burden of diarrheal diseases is closely associated with water, sanitation, and hygiene conditions, nutritional status, and the introduction of oral vaccines. Although rates have declined over time, the absolute burden remains high in lower-SDI G20 members, where pathogen profiles continue to evolve [10]. Therefore, effective diarrheal disease control cannot rely solely on clinical treatment but must also be grounded in improvements in water and sanitation infrastructure, nutritional support, rotavirus vaccination, strengthened primary-level rehydration capacity, and effective referral systems. In contrast, CNS infections are characterized by high death and severe long-term sequelae. Even with fewer cases than LRIs or diarrhea, meningitis and encephalitis may contribute disproportionately to death and DALYs. Studies of infectious encephalitis among Asian children demonstrate a strong inverse relationship with SDI and highlight lower-SDI regions as priority targets for prevention and control [13]. Accordingly, strengthening early diagnosis and treatment for suspected CNS infections, expanding access to laboratory-based pathogen detection, and establishing long-term follow-up systems for survivors are essential components of pediatric infectious disease management in G20 countries.

We also observed that the burden is disproportionately concentrated in younger age groups, particularly among infants and young children. This pattern is consistent with biological vulnerabilities such as immature immune system development, the waning of maternally derived antibodies, and the compounding effects of prematurity, low birth weight, and malnutrition. Considering both pathogen spectra and infection syndromes, prevention priorities should focus on invasive pneumococcal disease and rotavirus-associated diarrhea. Moreover, we identified a clear SDI gradient in death and DALY burdens. The near-perfect inverse correlation shown in Fig 6a–6b indicates that socioeconomic development is the dominant predictor of regional burden, explaining most of the observed variance. This implies that health sector interventions alone are insufficient. Broader development policies addressing education, income, and governance are prerequisite to sustainable burden reduction. For pneumococcal disease, regions with insufficient PCV coverage continue to bear higher burdens [18], while for rotavirus, vaccine introduction and coverage levels directly influence the magnitude of reductions in hospitalizations and death [21]. These findings suggest that public health policies should prioritize targeted investments aimed at reducing inequities by ensuring that cost-effective interventions—including vaccines, oral rehydration therapy, antibiotics, and pathogen diagnostics—are preferentially delivered to high-burden regions and the most vulnerable pediatric populations. Based on our findings, we propose four actionable priorities for G20 countries: (1) Prioritize PCV scale-up in lower-SDI G20 members, where pneumococcus remains the leading killer; (2) Combine rotavirus vaccination with improved ORS access to maximize diarrheal death reduction; (3) Strengthen surveillance for *Klebsiella pneumoniae*, particularly neonatal sepsis in lower-SDI G20 settings, given emerging antimicrobial resistance; (4) Accelerate *Shigella* vaccine development and deployment, as it ranks among the top five causes of diarrheal DALYs in G20 countries. These priorities are directly supported by the temporal trends **(**Fig 1a**–**1b), which show that pathogens with existing vaccines declined most steeply, while those lacking vaccines (e.g., *Klebsiella*, *Shigella*) persisted or increased, suggesting that vaccine development pipeline investment should target these remaining gaps.

This study provides an updated descriptive analysis of pediatric infectious disease burden in G20 countries from a “multi-pathogen–multi-syndrome” perspective, systematically comparing the attributable burden of 46 key pathogens within a unified analytical framework. This approach facilitates the identification of prevention priorities across pathogens and syndromes on a comparable scale. Furthermore, by utilizing the most recent GBD 2023 data cycle, our analysis provides updated evidence relevant to pediatric infectious disease prevention and control in the post-pandemic era within the G20. Stratified analyses across G20 member states and SDI levels further enable the identification of high-burden clusters, thereby offering practical guidance for resource allocation.

However, several limitations should be acknowledged. First, our findings reflect modeled estimates from GBD rather than primary observational data. GBD estimates rely on the integration of multiple data sources and the availability of surveillance systems; therefore, uncertainty may be greater in G20 members with limited monitoring capacity. Second, there is potential for misclassification or uncertainty in pathogen attribution, which must be critically emphasized. Pathogen-level attribution is model-dependent, co-infections are not well captured, our estimates differ from surveillance-based data, and misclassification across syndromes may occur (e.g., sepsis with meningitis may be undercounted). Third, aggregating all infants under 1 year into a single age group may oversubscribe morbidity and death to *Streptococcus pneumoniae* in G20-specific analyses, as pathogen etiology varies substantially across neonates (0–28 days), young infants (1– < 6 months), and older infants (6– < 12 months). Fourth, substantial variability in data quality exists across G20 member states. Although we examined differences across major countries, substantial heterogeneity within countries may be obscured by national averages, and high-income G20 members typically have more robust surveillance systems than lower-SDI members. Fifth, the ecological nature of our analysis limits causal inference. The observed associations between SDI and disease burden should be interpreted as descriptive patterns rather than causal relationships. Sixth, the methodological strength of this study should not be overclaimed; our analysis is a descriptive synthesis of GBD estimates rather than a methodological innovation. Moreover, formal pre-post COVID comparison (2019 vs. 2020–2023) was not performed due to potential surveillance disruptions during the acute pandemic phase.

## 5 Conclusions

Although the overall burden of pediatric infectious diseases in G20 countries has declined markedly since 1990, substantial burdens attributable to pathogens such as Streptococcus pneumoniae, rotavirus, and Klebsiella pneumoniae persist in lower-SDI member states. Moving forward, priority should be given to increasing coverage of pneumococcal conjugate vaccines and rotavirus vaccines in resource-limited G20 settings, strengthening pathogen surveillance, and improving access to timely diagnosis and treatment for severe infections in order to further reduce health inequalities.

## Supporting information

S1 Table Deaths and death rates for 46 pathogens among children in G20 countries aged 0–14 years, by infectious syndrome, 2023. (DOCX)

S2 Table Disability-adjusted life years (DALYs) and DALY rates for 46 pathogens among children in G20 countries aged 0–14 years, by infectious syndrome, 2023. (DOCX)

S3 Table Estimated annual percentage change (EAPC) in death(A) and DALY(B) rates for each pathogen among children in G20 countries aged 0–14 years, 1990–2023. (DOCX)
