# Supplementary material for: Burden of 46 key pathogens among children in G20 countries, 2023: A systematic analysis and regional comparison
Source: PLoS Negl Trop Dis. 2026 Sep 17;20(9):e0014723. doi: 10.1371/journal.pntd.0014723 (PMC13585249; doi:10.1371/journal.pntd.0014723)
Supplement: Supplementary file 1 [file pntd.0014723.s001.docx]

**S1 Table.** Deaths and death rates for 46 pathogens among children in G20 countries aged 0–14 years, by infectious syndrome, 2023.

| **Pathogen** | **All 3 infectious syndromes** | | **Lower respiratory infections** | | **Diarrheal diseases** | | **Meningitis** | |
| --- | --- | --- | --- | --- | --- | --- | --- | --- |
|  | **Death cases** | **Rate per 100,000 (95%UI)** | **Death cases** | **Rate per 100,000 (95%UI)** | **Death cases** | **Rate per 100,000 (95%UI)** | **Death cases** | **Rate per 100,000 (95%UI)** |
| Acinetobacter baumannii | 3548.357 (2897.650 - 4384.936) | 0.368 (0.300 - 0.454) | 3184.552 (2629.693 - 3871.428) | 0.330 (0.272 - 0.401) |  |  | 363.805 (267.957 - 513.508) | 0.038 (0.028 - 0.053) |
| Adenovirus | 16659.888 (9316.318 - 26158.864) | 1.725 (0.964 - 2.708) |  |  | 16659.888 (9316.318 - 26158.864) | 1.725 (0.964 - 2.708) |  |  |
| Aeromonas | 1811.544 (852.313 - 3429.583) | 0.188 (0.088 - 0.355) |  |  | 1811.544 (852.313 - 3429.583) | 0.188 (0.088 - 0.355) |  |  |
| Aspergillus spp. | 3040.424 (2391.468 - 3951.542) | 0.315 (0.248 - 0.409) | 3040.424 (2391.468 - 3951.542) | 0.315 (0.248 - 0.409) |  |  |  |  |
| Candida spp. | 762.760 (585.151 - 991.565) | 0.079 (0.061 - 0.103) |  |  |  |  | 762.760 (585.151 - 991.565) | 0.079 (0.061 - 0.103) |
| Campylobacter | 4977.026 (2271.335 - 9779.184) | 0.515 (0.235 - 1.012) |  |  | 4977.026 (2271.335 - 9779.184) | 0.515 (0.235 - 1.012) |  |  |
| Cholera | 3512.206 (2005.178 - 5462.903) | 0.364 (0.208 - 0.566) |  |  | 3512.206 (2005.178 - 5462.903) | 0.364 (0.208 - 0.566) |  |  |
| Clostridium difficile | 115.709 (81.444 - 170.709) | 0.012 (0.008 - 0.018) |  |  | 115.709 (81.444 - 170.709) | 0.012 (0.008 - 0.018) |  |  |
| Cryptosporidium | 5243.993 (2813.936 - 8846.671) | 0.543 (0.291 - 0.916) |  |  | 5243.993 (2813.936 - 8846.671) | 0.543 (0.291 - 0.916) |  |  |
| Chlamydia spp | 3104.440 (2440.525 - 3754.046) | 0.321 (0.253 - 0.389) | 3104.440 (2440.525 - 3754.046) | 0.321 (0.253 - 0.389) |  |  |  |  |
| Citrobacter spp. | 517.150 (371.620 - 696.213) | 0.054 (0.038 - 0.072) | 517.150 (371.620 - 696.213) | 0.054 (0.038 - 0.072) |  |  |  |  |
| Coagulase negative staphylococci | 597.237 (472.903 - 755.646) | 0.062 (0.049 - 0.078) |  |  |  |  | 597.237 (472.903 - 755.646) | 0.062 (0.049 - 0.078) |
| Enterobacter spp | 1370.242 (1101.366 - 1641.219) | 0.142 (0.114 - 0.170) | 1370.242 (1101.366 - 1641.219) | 0.142 (0.114 - 0.170) |  |  |  |  |
| Entamoeba | 4477.094 (1933.437 - 8405.939) | 0.463 (0.200 - 0.870) |  |  | 4477.094 (1933.437 - 8405.939) | 0.463 (0.200 - 0.870) |  |  |
| Escherichia coli | 10324.158 (8606.969 - 12023.763) | 1.069 (0.891 - 1.244) | 9975.323 (8325.659 - 11594.982) | 1.033 (0.862 - 1.200) |  |  | 348.835 (281.310 - 428.781) | 0.036 (0.029 - 0.044) |
| Enteropathogenic E coli | 5480.856 (2733.926 - 9622.492) | 0.567 (0.283 - 0.996) |  |  | 5480.856 (2733.926 - 9622.492) | 0.567 (0.283 - 0.996) |  |  |
| Enterotoxigenic E coli | 7068.311 (4182.515 - 11086.358) | 0.732 (0.433 - 1.148) |  |  | 7068.311 (4182.515 - 11086.358) | 0.732 (0.433 - 1.148) |  |  |
| Group A Streptococcus | 5176.424 (4421.127 - 5945.150) | 0.536 (0.458 - 0.615) | 5176.424 (4421.127 - 5945.150) | 0.536 (0.458 - 0.615) |  |  |  |  |
| Group B streptococcus | 3844.966 (3072.293 - 4809.740) | 0.398 (0.318 - 0.497) | 2647.609 (2156.676 - 3269.076) | 0.274 (0.223 - 0.338) |  |  | 1197.357 (915.617 - 1540.664) | 0.124 (0.095 - 0.159) |
| Haemophilus influenzae | 5430.254 (4474.221 - 6559.140) | 0.562 (0.464 - 0.679) | 4817.157 (3974.983 - 5768.294) | 0.499 (0.412 - 0.597) |  |  | 613.097 (499.238 - 790.846) | 0.063 (0.052 - 0.082) |
| Influenza | 4629.421 (3274.936 - 6671.561) | 0.479 (0.339 - 0.691) | 4629.421 (3274.936 - 6671.561) | 0.479 (0.339 - 0.691) |  |  |  |  |
| Klebsiella pneumoniae | 21481.923 (17948.480 - 24660.640) | 2.224 (1.859 - 2.553) | 20930.093 (17508.200 - 23940.873) | 2.167 (1.813 - 2.478) |  |  | 551.830 (440.280 - 719.767) | 0.057 (0.046 - 0.075) |
| Legionella spp | 265.859 (195.161 - 352.829) | 0.028 (0.020 - 0.037) | 265.859 (195.161 - 352.829) | 0.028 (0.020 - 0.037) |  |  |  |  |
| Listeria monocytogenes | 549.291 (433.630 - 708.539) | 0.057 (0.045 - 0.073) |  |  |  |  | 549.291 (433.630 - 708.539) | 0.057 (0.045 - 0.073) |
| Morganella spp. | 215.868 (149.575 - 319.119) | 0.022 (0.015 - 0.033) | 215.868 (149.575 - 319.119) | 0.022 (0.015 - 0.033) |  |  |  |  |
| Mycoplasma | 5534.160 (4595.089 - 6486.606) | 0.573 (0.476 - 0.672) | 5534.160 (4595.089 - 6486.606) | 0.573 (0.476 - 0.672) |  |  |  |  |
| Neisseria meningitidis | 2002.392 (1627.658 - 2585.101) | 0.207 (0.169 - 0.268) |  |  |  |  | 2002.392 (1627.658 - 2585.101) | 0.207 (0.169 - 0.268) |
| Non-polio enteroviruses | 852.160 (679.659 - 1090.833) | 0.088 (0.070 - 0.113) |  |  |  |  | 852.160 (679.659 - 1090.833) | 0.088 (0.070 - 0.113) |
| Non-typhoidal Salmonella | 1897.566 (348.139 - 3914.403) | 0.196 (0.036 - 0.405) |  |  | 1897.566 (348.139 - 3914.403) | 0.196 (0.036 - 0.405) |  |  |
| Norovirus | 4490.122 (1547.519 - 8085.838) | 0.465 (0.160 - 0.837) |  |  | 4490.122 (1547.519 - 8085.838) | 0.465 (0.160 - 0.837) |  |  |
| Other Acinetobacter species | 835.331 (672.974 - 1038.489) | 0.086 (0.070 - 0.108) | 835.331 (672.974 - 1038.489) | 0.086 (0.070 - 0.108) |  |  |  |  |
| Other bacterial and viral pathogens | 4777.301 (3589.416 - 5961.323) | 0.495 (0.371 - 0.617) | 4328.098 (3239.032 - 5332.997) | 0.448 (0.335 - 0.552) |  |  | 449.203 (350.384 - 628.326) | 0.047 (0.036 - 0.065) |
| Other fungi | 2300.616 (1709.626 - 3106.392) | 0.238 (0.177 - 0.322) | 1596.179 (1204.994 - 2106.441) | 0.165 (0.125 - 0.218) |  |  | 704.437 (504.632 - 999.951) | 0.073 (0.052 - 0.104) |
| Other gram-negative bacteria | 3956.275 (3353.448 - 4619.492) | 0.410 (0.347 - 0.478) | 3956.275 (3353.448 - 4619.492) | 0.410 (0.347 - 0.478) |  |  |  |  |
| Other Klebsiella species | 1448.221 (1189.795 - 1675.920) | 0.150 (0.123 - 0.174) | 1448.221 (1189.795 - 1675.920) | 0.150 (0.123 - 0.174) |  |  |  |  |
| Other streptococcus species | 623.664 (506.581 - 792.540) | 0.065 (0.052 - 0.082) |  |  |  |  | 623.664 (506.581 - 792.540) | 0.065 (0.052 - 0.082) |
| Other viral etiologies of meningitis | 842.660 (691.687 - 1112.698) | 0.087 (0.072 - 0.115) |  |  |  |  | 842.660 (691.687 - 1112.698) | 0.087 (0.072 - 0.115) |
| Other Mycobacterium species (non-TB, non-Leprosy) | 8503.469 (6009.413 - 12094.369) | 0.880 (0.622 - 1.252) | 8503.469 (6009.413 - 12094.369) | 0.880 (0.622 - 1.252) |  |  |  |  |
| Proteus spp. | 292.938 (241.724 - 366.551) | 0.030 (0.025 - 0.038) | 292.938 (241.724 - 366.551) | 0.030 (0.025 - 0.038) |  |  |  |  |
| Pseudomonas aeruginosa | 13010.717 (11023.146 - 15006.662) | 1.347 (1.141 - 1.553) | 12564.568 (10668.815 - 14425.663) | 1.301 (1.104 - 1.493) |  |  | 446.149 (354.331 - 580.999) | 0.046 (0.037 - 0.060) |
| Respiratory syncytial virus | 5373.225 (3715.553 - 7862.622) | 0.556 (0.385 - 0.814) | 5373.225 (3715.553 - 7862.622) | 0.556 (0.385 - 0.814) |  |  |  |  |
| Rotavirus | 16125.003 (11124.277 - 22903.026) | 1.669 (1.152 - 2.371) |  |  | 16125.003 (11124.277 - 22903.026) | 1.669 (1.152 - 2.371) |  |  |
| Serratia spp. | 713.644 (577.628 - 852.118) | 0.074 (0.060 - 0.088) | 713.644 (577.628 - 852.118) | 0.074 (0.060 - 0.088) |  |  |  |  |
| Staphylococcus aureus | 10500.983 (8993.304 - 11898.438) | 1.088 (0.931 - 1.232) | 10050.714 (8620.312 - 11341.994) | 1.041 (0.892 - 1.174) |  |  | 450.269 (372.992 - 556.444) | 0.047 (0.039 - 0.058) |
| Streptococcus pneumoniae | 66394.314 (56369.219 - 76988.586) | 6.873 (5.836 - 7.970) | 63822.665 (54264.434 - 73576.289) | 6.607 (5.618 - 7.617) |  |  | 2571.649 (2104.785 - 3412.297) | 0.266 (0.218 - 0.353) |
| Shigella | 9736.276 (5712.697 - 15428.745) | 1.008 (0.591 - 1.597) |  |  | 9736.276 (5712.697 - 15428.745) | 1.008 (0.591 - 1.597) |  |  |
