# Supplementary material for: Burden of 46 key pathogens among children in G20 countries, 2023: A systematic analysis and regional comparison
Source: PLoS Negl Trop Dis. 2026 Sep 17;20(9):e0014723. doi: 10.1371/journal.pntd.0014723 (PMC13585249; doi:10.1371/journal.pntd.0014723)
Supplement: Supplementary file 2 [file pntd.0014723.s002.docx]

**S2 Table.**  Disability-adjusted life years (DALYs) and DALY rates for 46 pathogens among children in G20 countries aged 0–14 years, by infectious syndrome, 2023.

| **Pathogen** | **All 3 infectious syndromes** | | **Lower respiratory infections** | | **Diarrheal diseases** | | **Meningitis** | |
| --- | --- | --- | --- | --- | --- | --- | --- | --- |
|  | **DALYs cases (95%UI)** | **Rate per 100,000 (95%UI)** | **DALYs cases (95%UI)** | **Rate per 100,000 (95%UI)** | **DALYs cases (95%UI)** | **Rate per 100,000 (95%UI)** | **DALYs cases (95%UI)** | **Rate per 100,000 (95%UI)** |
| Acinetobacter baumannii | 315700.878 (257419.109 - 390506.137) | 32.683 (26.650 - 40.428) | 283352.465 (233689.549 - 344845.845) | 29.334 (24.193 - 35.700) |  |  | 32348.413 (23729.560 - 45660.292) | 3.349 (2.457 - 4.727) |
| Adenovirus | 1610937.727 (977911.674 - 2480813.727) | 166.774 (101.239 - 256.828) |  |  | 1610937.727 (977911.674 - 2480813.727) | 166.774 (101.239 - 256.828) |  |  |
| Aeromonas | 175170.220 (85489.239 - 318279.532) | 18.135 (8.850 - 32.950) |  |  | 175170.220 (85489.239 - 318279.532) | 18.135 (8.850 - 32.950) |  |  |
| Aspergillus spp. | 268074.931 (209740.536 - 350333.133) | 27.753 (21.714 - 36.269) | 268074.931 (209740.536 - 350333.133) | 27.753 (21.714 - 36.269) |  |  |  |  |
| Candida spp. | 68262.668 (52636.309 - 88222.950) | 7.067 (5.449 - 9.133) |  |  |  |  | 68262.668 (52636.309 - 88222.950) | 7.067 (5.449 - 9.133) |
| Campylobacter | 517335.856 (260532.136 - 969584.641) | 53.558 (26.972 - 100.377) |  |  | 517335.856 (260532.136 - 969584.641) | 53.558 (26.972 - 100.377) |  |  |
| Cholera | 298664.049 (171350.771 - 468780.755) | 30.919 (17.739 - 48.531) |  |  | 298664.049 (171350.771 - 468780.755) | 30.919 (17.739 - 48.531) |  |  |
| Clostridium difficile | 9721.637 (6932.979 - 14094.782) | 1.006 (0.718 - 1.459) |  |  | 9721.637 (6932.979 - 14094.782) | 1.006 (0.718 - 1.459) |  |  |
| Cryptosporidium | 492729.502 (267120.397 - 819288.296) | 51.010 (27.654 - 84.817) |  |  | 492729.502 (267120.397 - 819288.296) | 51.010 (27.654 - 84.817) |  |  |
| Chlamydia spp | 278627.538 (219143.909 - 337140.586) | 28.845 (22.687 - 34.903) | 278627.538 (219143.909 - 337140.586) | 28.845 (22.687 - 34.903) |  |  |  |  |
| Citrobacter spp. | 45981.819 (32947.075 - 61992.056) | 4.760 (3.411 - 6.418) | 45981.819 (32947.075 - 61992.056) | 4.760 (3.411 - 6.418) |  |  |  |  |
| Coagulase negative staphylococci | 53180.754 (42095.112 - 67570.291) | 5.506 (4.358 - 6.995) |  |  |  |  | 53180.754 (42095.112 - 67570.291) | 5.506 (4.358 - 6.995) |
| Enterobacter spp | 122557.028 (98522.278 - 146830.360) | 12.688 (10.200 - 15.201) | 122557.028 (98522.278 - 146830.360) | 12.688 (10.200 - 15.201) |  |  |  |  |
| Entamoeba | 423767.810 (191205.898 - 766817.420) | 43.871 (19.795 - 79.385) |  |  | 423767.810 (191205.898 - 766817.420) | 43.871 (19.795 - 79.385) |  |  |
| Escherichia coli | 921802.084 (768400.279 - 1074963.653) | 95.430 (79.549 - 111.183) | 890560.740 (743286.430 - 1035734.464) | 92.196 (76.949 - 107.225) |  |  | 31241.344 (25113.849 - 38229.189) | 3.234 (2.600 - 3.958) |
| Enteropathogenic E coli | 516592.333 (263322.019 - 890236.499) | 53.481 (27.261 - 92.162) |  |  | 516592.333 (263322.019 - 890236.499) | 53.481 (27.261 - 92.162) |  |  |
| Enterotoxigenic E coli | 704750.874 (436825.062 - 1089053.840) | 72.960 (45.223 - 112.745) |  |  | 704750.874 (436825.062 - 1089053.840) | 72.960 (45.223 - 112.745) |  |  |
| Group A Streptococcus | 462288.716 (394754.318 - 531291.812) | 47.859 (40.867 - 55.002) | 462288.716 (394754.318 - 531291.812) | 47.859 (40.867 - 55.002) |  |  |  |  |
| Group B streptococcus | 344206.018 (275118.247 - 440871.897) | 35.634 (28.482 - 45.607) | 236832.321 (192964.291 - 292746.236) | 24.518 (19.977 - 30.307) |  |  | 107373.697 (82153.956 - 138125.661) | 11.116 (8.505 - 14.300) |
| Haemophilus influenzae | 487923. - |  | 432463.358 (357290.359 - 518997.790) | 44.771 (36.989 - 53.730) |  |  | 55459.639 (45356.067 - 71059.520) | 5.742 (4.696 - 7.356) |
| Influenza | 412606.700 (291624.222 - 595118.680) | 42.715 (30.191 - 61.610) | 412606.700 (291624.222 - 595118.680) | 42.715 (30.191 - 61.610) |  |  |  |  |
| Klebsiella pneumoniae | 1917687.981 (1600781.719 - 2202024.594) | 198.531 (165.722 - 227.962) | 1868273.365 (1561100.746 - 2137476.052) | 193.415 (161.614 - 221.284) |  |  | 49414.616 (39680.973 - 64508.542) | 5.116 (4.108 - 6.678) |
| Legionella spp | 22960.358 (16709.054 - 30743.784) | 2.377 (1.730 - 3.183) | 22960.358 (16709.054 - 30743.784) | 2.377 (1.730 - 3.183) |  |  |  |  |
| Listeria monocytogenes | 48799.701 (38622.724 - 62978.000) | 5.052 (3.998 - 6.520) |  |  |  |  | 48799.701 (38622.724 - 62978.000) | 5.052 (3.998 - 6.520) |
| Morganella spp. | 19198.890 (13272.376 - 28465.378) | 1.988 (1.374 - 2.947) | 19198.890 (13272.376 - 28465.378) | 1.988 (1.374 - 2.947) |  |  |  |  |
| Mycoplasma | 492795.721 (409215.168 - 577595.468) | 51.017 (42.364 - 59.796) | 492795.721 (409215.168 - 577595.468) | 51.017 (42.364 - 59.796) |  |  |  |  |
| Neisseria meningitidis | 178798.753 (146252.724 - 230341.893) | 18.510 (15.141 - 23.846) |  |  |  |  | 178798.753 (146252.724 - 230341.893) | 18.510 (15.141 - 23.846) |
| Non-polio enteroviruses | 91638.993 (74950.994 - 115637.829) | 9.487 (7.759 - 11.972) |  |  |  |  | 91638.993 (74950.994 - 115637.829) | 9.487 (7.759 - 11.972) |
| Non-typhoidal Salmonella | 182154.925 (46726.031 - 362659.350) | 18.858 (4.837 - 37.545) |  |  | 182154.925 (46726.031 - 362659.350) | 18.858 (4.837 - 37.545) |  |  |
| Norovirus | 501909.945 (219715.909 - 833746.197) | 51.961 (22.746 - 86.314) |  |  | 501909.945 (219715.909 - 833746.197) | 51.961 (22.746 - 86.314) |  |  |
| Other Acinetobacter species | 74183.287 (59637.314 - 92243.946) | 7.680 (6.174 - 9.550) | 74183.287 (59637.314 - 92243.946) | 7.680 (6.174 - 9.550) |  |  |  |  |
| Other bacterial and viral pathogens | 428230.207 (321796.949 - 534825.100) | 44.333 (33.314 - 55.368) | 386597.660 (288838.107 - 476893.848) | 40.023 (29.902 - 49.371) |  |  | 41632.547 (32958.842 - 57931.252) | 4.310 (3.412 - 5.997) |
| Other fungi | 205255.921 (152581.729 - 276937.101) | 21.249 (15.796 - 28.670) | 142073.847 (107125.089 - 187261.302) | 14.708 (11.090 - 19.386) |  |  | 63182.074 (45456.640 - 89675.799) | 6.541 (4.706 - 9.284) |
| Other gram-negative bacteria | 351270.955 (297987.260 - 410481.052) | 36.366 (30.849 - 42.495) | 351270.955 (297987.260 - 410481.052) | 36.366 (30.849 - 42.495) |  |  |  |  |
| Other Klebsiella species | 128954.659 (105848.390 - 149402.899) | 13.350 (10.958 - 15.467) | 128954.659 (105848.390 - 149402.899) | 13.350 (10.958 - 15.467) |  |  |  |  |
| Other streptococcus species | 55406.274 (44764.235 - 70820.315) | 5.736 (4.634 - 7.332) |  |  |  |  | 55406.274 (44764.235 - 70820.315) | 5.736 (4.634 - 7.332) |
| Other viral etiologies of meningitis | 77073.466 (64134.022 - 100083.771) | 7.979 (6.640 - 10.361) |  |  |  |  | 77073.466 (64134.022 - 100083.771) | 7.979 (6.640 - 10.361) |
| Other Mycobacterium species (non-TB, non-Leprosy) | 756015.578 (532823.414 - 1078077.532) | 78.267 (55.161 - 111.609) | 756015.578 (532823.414 - 1078077.532) | 78.267 (55.161 - 111.609) |  |  |  |  |
| Proteus spp. | 25894.116 (21297.147 - 32484.423) | 2.681 (2.205 - 3.363) | 25894.116 (21297.147 - 32484.423) | 2.681 (2.205 - 3.363) |  |  |  |  |
| Pseudomonas aeruginosa | 1160415.500 (982146.395 - 1339291.581) | 120.133 (101.681 - 138.651) | 1120336.794 (951393.589 - 1286881.461) | 115.984 (98.494 - 133.225) |  |  | 40078.706 (31752.806 - 52410.120) | 4.149 (3.287 - 5.426) |
| Respiratory syncytial virus | 483161.884 (334432.415 - 707829.900) | 50.020 (34.622 - 73.279) | 483161.884 (334432.415 - 707829.900) | 50.020 (34.622 - 73.279) |  |  |  |  |
| Rotavirus | 1614105.070 (1124262.092 - 2248233.149) | 167.102 (116.390 - 232.750) |  |  | 1614105.070 (1124262.092 - 2248233.149) | 167.102 (116.390 - 232.750) |  |  |
| Serratia spp. | 63779.361 (51543.622 - 76223.208) | 6.603 (5.336 - 7.891) | 63779.361 (51543.622 - 76223.208) | 6.603 (5.336 - 7.891) |  |  |  |  |
| Staphylococcus aureus | 932719.870 (798757.060 - 1057063.416) | 96.561 (82.692 - 109.434) | 892646.865 (765439.141 - 1007243.871) | 92.412 (79.243 - 104.276) |  |  | 40073.005 (33317.919 - 49819.545) | 4.149 (3.449 - 5.158) |
| Streptococcus pneumoniae | 5901914.988 (5011319.774 - 6845714.670) | 610.991 (518.786 - 708.709) | 5674640.821 (4824710.057 - 6544160.873) | 587.472 (499.482 - 677.490) |  |  | 227274.167 (186609.717 - 301553.797) | 23.529 (19.319 - 31.219) |
| Shigella | 986660.385 (603220.226 - 1537554.390) | 102.145 (62.449 - 159.177) |  |  | 986660.385 (603220.226 - 1537554.390) | 102.145 (62.449 - 159.177) |  |  |
