# Supplementary material for: Burden of 46 key pathogens among children in G20 countries, 2023: A systematic analysis and regional comparison
Source: PLoS Negl Trop Dis. 2026 Sep 17;20(9):e0014723. doi: 10.1371/journal.pntd.0014723 (PMC13585249; doi:10.1371/journal.pntd.0014723)
Supplement: Supplementary file 3 [file pntd.0014723.s003.docx]

**S3 Table A. Deaths and estimated annual percentage change for 46 key pathogens among children in G20 countries aged 0–14 years, 1990 and 2023.**

| **Index** | **level** | **1990** | | **2023** | | **1990–2023** | |
| --- | --- | --- | --- | --- | --- | --- | --- |
|  |  | **Number (95 %UI)** | **Rate per 100,000(95%UI)** | **Number (95 %UI)** | **Rate per 100,000(95%UI)** | **Rate change** | **Rate EAPC(95 %CI)** |
| Diarrheal diseases | Non-typhoidal Salmonella | 27515.051(4497.516-57760.518) | 2.693(0.440-5.654) | 1897.566(348.139-3914.403) | 0.196(0.036-0.405) | -0.927(-0.948--0.896) | -7.65(-7.87,-7.43) |
| Diarrheal diseases | Shigella | 140920.149(84929.428-235842.437) | 13.794(8.313-23.086) | 9736.276(5712.697-15428.745) | 1.008(0.591-1.597) | -0.927(-0.947--0.905) | -7.50(-7.91,-7.08) |
| Diarrheal diseases | Enteropathogenic E coli | 64582.587(33913.223-123299.353) | 6.322(3.320-12.069) | 5480.856(2733.926-9622.492) | 0.567(0.283-0.996) | -0.910(-0.936--0.874) | -6.77(-7.34,-6.20) |
| Diarrheal diseases | Enterotoxigenic E coli | 92888.908(55804.358-140352.422) | 9.093(5.462-13.739) | 7068.311(4182.515-11086.358) | 0.732(0.433-1.148) | -0.920(-0.947--0.890) | -7.46(-7.90,-7.02) |
| Diarrheal diseases | Campylobacter | 80990.076(35602.882-153130.442) | 7.928(3.485-14.989) | 4977.026(2271.335-9779.184) | 0.515(0.235-1.012) | -0.935(-0.955--0.911) | -7.76(-8.20,-7.31) |
| Diarrheal diseases | Entamoeba | 58698.132(25022.159-108200.386) | 5.746(2.449-10.591) | 4477.094(1933.437-8405.939) | 0.463(0.200-0.870) | -0.919(-0.943--0.891) | -7.38(-7.59,-7.17) |
| Diarrheal diseases | Cryptosporidium | 73734.235(41853.244-129410.644) | 7.218(4.097-12.668) | 5243.993(2813.936-8846.671) | 0.543(0.291-0.916) | -0.925(-0.946--0.899) | -6.88(-7.30,-6.46) |
| Diarrheal diseases | Rotavirus | 286834.995(218921.208-376401.283) | 28.077(21.429-36.844) | 16125.003(11124.277-22903.026) | 1.669(1.152-2.371) | -0.941(-0.958--0.920) | -7.64(-8.20,-7.07) |
| Diarrheal diseases | Aeromonas | 31321.905(14713.023-57902.849) | 3.066(1.440-5.668) | 1811.544(852.313-3429.583) | 0.188(0.088-0.355) | -0.939(-0.959--0.916) | -8.12(-8.40,-7.85) |
| Diarrheal diseases | Clostridium difficile | 91.122(57.413-133.587) | 0.009(0.006-0.013) | 115.709(81.444-170.709) | 0.012(0.008-0.018) | 0.343(0.089-0.652) | 1.22(0.92,1.51) |
| Diarrheal diseases | Norovirus | 70373.975(27137.803-124383.823) | 6.889(2.656-12.175) | 4490.122(1547.519-8085.838) | 0.465(0.160-0.837) | -0.933(-0.956--0.908) | -7.91(-8.17,-7.66) |
| Diarrheal diseases | Adenovirus | 208859.313(115481.667-331427.690) | 20.444(11.304-32.442) | 16659.888(9316.318-26158.864) | 1.725(0.964-2.708) | -0.916(-0.940--0.880) | -7.02(-7.16,-6.88) |
| Diarrheal diseases | Cholera | 26610.064(15456.506-45637.622) | 2.605(1.513-4.467) | 3512.206(2005.178-5462.903) | 0.364(0.208-0.566) | -0.860(-0.914--0.771) | -6.09(-6.47,-5.71) |
| Lower respiratory infections | Influenza | 44146.848(37556.342-50816.646) | 4.321(3.676-4.974) | 4629.421(3274.936-6671.561) | 0.479(0.339-0.691) | -0.889(-0.924--0.831) | -5.94(-6.78,-5.10) |
| Lower respiratory infections | Respiratory syncytial virus | 45619.852(38838.914-53188.023) | 4.466(3.802-5.206) | 5373.225(3715.553-7862.622) | 0.556(0.385-0.814) | -0.875(-0.917--0.804) | -5.55(-6.37,-4.72) |
| Lower respiratory infections | Group B streptococcus | 9467.968(7380.388-12222.350) | 0.927(0.722-1.196) | 2647.609(2156.676-3269.076) | 0.274(0.223-0.338) | -0.704(-0.755--0.644) | -4.10(-4.31,-3.90) |
| Lower respiratory infections | Acinetobacter baumannii | 18249.783(15566.680-21533.447) | 1.786(1.524-2.108) | 3184.552(2629.693-3871.428) | 0.330(0.272-0.401) | -0.815(-0.846--0.780) | -5.42(-5.66,-5.18) |
| Lower respiratory infections | Chlamydia spp | 16625.167(13410.127-20561.354) | 1.627(1.313-2.013) | 3104.440(2440.525-3754.046) | 0.321(0.253-0.389) | -0.803(-0.835--0.762) | -5.29(-5.48,-5.09) |
| Lower respiratory infections | Enterobacter spp | 4109.831(3512.374-4908.911) | 0.402(0.344-0.481) | 1370.242(1101.366-1641.219) | 0.142(0.114-0.170) | -0.647(-0.708--0.581) | -3.53(-3.73,-3.33) |
| Lower respiratory infections | Escherichia coli | 31913.442(27166.316-37708.913) | 3.124(2.659-3.691) | 9975.323(8325.659-11594.982) | 1.033(0.862-1.200) | -0.669(-0.726--0.611) | -3.72(-3.91,-3.52) |
| Lower respiratory infections | Haemophilus influenzae | 21374.390(17840.964-25182.078) | 2.092(1.746-2.465) | 4817.157(3974.983-5768.294) | 0.499(0.412-0.597) | -0.762(-0.801--0.713) | -4.66(-4.89,-4.43) |
| Lower respiratory infections | Klebsiella pneumoniae | 94901.824(81899.693-111136.952) | 9.290(8.017-10.879) | 20930.093(17508.200-23940.873) | 2.167(1.813-2.478) | -0.767(-0.805--0.721) | -4.76(-4.95,-4.58) |
| Lower respiratory infections | Legionella spp | 149.138(98.593-214.317) | 0.015(0.010-0.021) | 265.859(195.161-352.829) | 0.028(0.020-0.037) | 0.885(0.632-1.276) | 2.00(1.65,2.35) |
| Lower respiratory infections | Mycoplasma | 25050.755(21065.490-28999.236) | 2.452(2.062-2.839) | 5534.160(4595.089-6486.606) | 0.573(0.476-0.672) | -0.766(-0.802--0.723) | -4.74(-4.93,-4.55) |
| Lower respiratory infections | Other bacterial and viral pathogens | 15154.661(12135.331-18592.475) | 1.483(1.188-1.820) | 4328.098(3239.032-5332.997) | 0.448(0.335-0.552) | -0.698(-0.755--0.641) | -4.02(-4.21,-3.84) |
| Lower respiratory infections | Pseudomonas aeruginosa | 48650.797(42144.328-55904.921) | 4.762(4.125-5.472) | 12564.568(10668.815-14425.663) | 1.301(1.104-1.493) | -0.727(-0.772--0.681) | -4.28(-4.46,-4.10) |
| Lower respiratory infections | Staphylococcus aureus | 27760.233(24177.752-31863.236) | 2.717(2.367-3.119) | 10050.714(8620.312-11341.994) | 1.041(0.892-1.174) | -0.617(-0.680--0.553) | -3.23(-3.42,-3.05) |
| Lower respiratory infections | Streptococcus pneumoniae | 463524.163(397389.159-529642.672) | 45.373(38.899-51.845) | 63822.665(54264.434-73576.289) | 6.607(5.618-7.617) | -0.854(-0.881--0.826) | -6.00(-6.34,-5.67) |
| Lower respiratory infections | Aspergillus spp. | 10402.368(8104.526-13321.690) | 1.018(0.793-1.304) | 3040.424(2391.468-3951.542) | 0.315(0.248-0.409) | -0.691(-0.741--0.634) | -3.89(-4.07,-3.70) |
| Lower respiratory infections | Other Acinetobacter species | 3897.954(3076.383-4783.377) | 0.382(0.301-0.468) | 835.331(672.974-1038.489) | 0.086(0.070-0.108) | -0.773(-0.810--0.730) | -4.84(-5.02,-4.65) |
| Lower respiratory infections | Citrobacter spp. | 1144.603(851.854-1548.597) | 0.112(0.083-0.152) | 517.150(371.620-696.213) | 0.054(0.038-0.072) | -0.522(-0.607--0.428) | -2.62(-2.82,-2.43) |
| Lower respiratory infections | Other fungi | 6787.783(5307.877-8622.272) | 0.664(0.520-0.844) | 1596.179(1204.994-2106.441) | 0.165(0.125-0.218) | -0.751(-0.791--0.705) | -4.58(-4.80,-4.37) |
| Lower respiratory infections | Other gram-negative bacteria | 17502.274(15112.294-20358.795) | 1.713(1.479-1.993) | 3956.275(3353.448-4619.492) | 0.410(0.347-0.478) | -0.761(-0.798--0.717) | -4.65(-4.84,-4.47) |
| Lower respiratory infections | Other Klebsiella species | 4249.203(3575.750-5068.333) | 0.416(0.350-0.496) | 1448.221(1189.795-1675.920) | 0.150(0.123-0.174) | -0.640(-0.698--0.576) | -3.45(-3.65,-3.26) |
| Lower respiratory infections | Morganella spp. | 877.273(606.249-1255.202) | 0.086(0.059-0.123) | 215.868(149.575-319.119) | 0.022(0.015-0.033) | -0.740(-0.786--0.687) | -4.44(-4.62,-4.26) |
| Lower respiratory infections | Other Mycobacterium species (non-TB, non-Leprosy) | 27948.995(19856.308-39042.704) | 2.736(1.944-3.822) | 8503.469(6009.413-12094.369) | 0.880(0.622-1.252) | -0.678(-0.735--0.620) | -3.84(-4.03,-3.65) |
| Lower respiratory infections | Proteus spp. | 787.964(641.797-987.185) | 0.077(0.063-0.097) | 292.938(241.724-366.551) | 0.030(0.025-0.038) | -0.607(-0.663--0.533) | -3.22(-3.41,-3.03) |
| Lower respiratory infections | Serratia spp. | 1987.302(1684.721-2353.428) | 0.195(0.165-0.230) | 713.644(577.628-852.118) | 0.074(0.060-0.088) | -0.620(-0.685--0.551) | -3.32(-3.54,-3.10) |
| Lower respiratory infections | Group A Streptococcus | 22741.408(18803.376-26702.987) | 2.226(1.841-2.614) | 5176.424(4421.127-5945.150) | 0.536(0.458-0.615) | -0.759(-0.800--0.715) | -4.61(-4.84,-4.38) |
| Meningitis | Escherichia coli | 2536.500(1885.172-3186.801) | 0.248(0.185-0.312) | 348.835(281.310-428.781) | 0.036(0.029-0.044) | -0.855(-0.888--0.808) | -5.86(-6.15,-5.58) |
| Meningitis | Haemophilus influenzae | 14184.496(10548.490-18035.642) | 1.388(1.033-1.765) | 613.097(499.238-790.846) | 0.063(0.052-0.082) | -0.954(-0.967--0.930) | -9.06(-9.98,-8.14) |
| Meningitis | Klebsiella pneumoniae | 5325.645(3958.137-6775.058) | 0.521(0.387-0.663) | 551.830(440.280-719.767) | 0.057(0.046-0.075) | -0.890(-0.918--0.850) | -6.69(-7.03,-6.36) |
| Meningitis | Listeria monocytogenes | 4418.385(3210.144-5752.156) | 0.432(0.314-0.563) | 549.291(433.630-708.539) | 0.057(0.045-0.073) | -0.869(-0.902--0.814) | -6.14(-6.44,-5.84) |
| Meningitis | Other bacterial and viral pathogens | 4459.357(3100.263-6028.636) | 0.437(0.303-0.590) | 449.203(350.384-628.326) | 0.047(0.036-0.065) | -0.893(-0.922--0.849) | -6.76(-7.10,-6.42) |
| Meningitis | Pseudomonas aeruginosa | 4095.690(2985.593-5247.437) | 0.401(0.292-0.514) | 446.149(354.331-580.999) | 0.046(0.037-0.060) | -0.885(-0.915--0.837) | -6.54(-6.87,-6.21) |
| Meningitis | Staphylococcus aureus | 2960.583(2340.555-3639.977) | 0.290(0.229-0.356) | 450.269(372.992-556.444) | 0.047(0.039-0.058) | -0.839(-0.873--0.784) | -5.54(-5.81,-5.28) |
| Meningitis | Streptococcus pneumoniae | 18300.658(13763.262-22416.008) | 1.791(1.347-2.194) | 2571.649(2104.785-3412.297) | 0.266(0.218-0.353) | -0.851(-0.889--0.788) | -5.71(-5.98,-5.44) |
| Meningitis | Neisseria meningitidis | 22527.525(17148.814-27871.701) | 2.205(1.679-2.728) | 2002.392(1627.658-2585.101) | 0.207(0.169-0.268) | -0.906(-0.930--0.871) | -7.24(-7.66,-6.82) |
| Meningitis | Other fungi | 5953.672(4014.196-8632.775) | 0.583(0.393-0.845) | 704.437(504.632-999.951) | 0.073(0.052-0.104) | -0.875(-0.905--0.826) | -6.28(-6.60,-5.96) |
| Meningitis | Candida spp. | 7069.111(5055.585-9313.989) | 0.692(0.495-0.912) | 762.760(585.151-991.565) | 0.079(0.061-0.103) | -0.886(-0.918--0.832) | -6.54(-6.86,-6.21) |
| Meningitis | Coagulase negative staphylococci | 6326.210(4964.082-7927.793) | 0.619(0.486-0.776) | 597.237(472.903-755.646) | 0.062(0.049-0.078) | -0.900(-0.927--0.858) | -6.98(-7.33,-6.64) |
| Meningitis | Non-polio enteroviruses | 11783.733(9215.163-14846.805) | 1.153(0.902-1.453) | 852.160(679.659-1090.833) | 0.088(0.070-0.113) | -0.924(-0.944--0.893) | -7.72(-8.19,-7.24) |
| Meningitis | Other streptococcus species | 4972.343(3659.880-6212.672) | 0.487(0.358-0.608) | 623.664(506.581-792.540) | 0.065(0.052-0.082) | -0.867(-0.899--0.816) | -6.11(-6.40,-5.81) |
| Meningitis | Other viral etiologies of meningitis | 7921.126(6248.230-9931.812) | 0.775(0.612-0.972) | 842.660(691.687-1112.698) | 0.087(0.072-0.115) | -0.887(-0.916--0.845) | -6.62(-6.95,-6.29) |
| Meningitis | Group B streptococcus | 8517.645(6603.426-10771.699) | 0.834(0.646-1.054) | 1197.357(915.617-1540.664) | 0.124(0.095-0.159) | -0.851(-0.893--0.771) | -5.67(-5.93,-5.41) |
| Meningitis | Acinetobacter baumannii | 3440.859(2336.435-4719.833) | 0.337(0.229-0.462) | 363.805(267.957-513.508) | 0.038(0.028-0.053) | -0.888(-0.917--0.845) | -6.63(-6.97,-6.30) |

Abbreviations: PC, percentage change; EAPC, estimated annual percentage change; UI, uncertainty interval.

**S3 Table B. DALYs and estimated annual percentage change for 46 key pathogens among children in G20 countries aged 0–14 years, 1990 and 2023.**

| **Index** | **level** | **1990** | | **2023** | | **1990–2023** | |
| --- | --- | --- | --- | --- | --- | --- | --- |
|  |  | **Number (95 %UI)** | **Rate per 100,000(95%UI)** | **Number (95 %UI)** | **Rate per 100,000(95%UI)** | **Rate change** | **Rate EAPC(95 %CI)** |
| Diarrheal diseases | Cholera | 2275714.329(1329053.896-3875001.192) | 222.761(130.096-379.309) | 298664.049(171350.771-468780.755) | 30.919(17.739-48.531) | -0.861(-0.915--0.771) | -6.12(-6.50,-5.73) |
| Diarrheal diseases | Non-typhoidal Salmonella | 2471512.959(446002.522-5145126.302) | 241.927(43.657-503.637) | 182154.925(46726.031-362659.350) | 18.858(4.837-37.545) | -0.922(-0.944--0.878) | -7.50(-7.71,-7.30) |
| Diarrheal diseases | Shigella | 12703559.606(7679590.874-21221346.970) | 1243.502(751.725-2077.276) | 986660.385(603220.226-1537554.390) | 102.145(62.449-159.177) | -0.918(-0.941--0.892) | -7.25(-7.64,-6.86) |
| Diarrheal diseases | Enteropathogenic E coli | 5813908.007(3080536.448-11080379.073) | 569.101(301.542-1084.615) | 516592.333(263322.019-890236.499) | 53.481(27.261-92.162) | -0.906(-0.933--0.869) | -6.68(-7.24,-6.12) |
| Diarrheal diseases | Enterotoxigenic E coli | 8407323.335(5052636.425-12655852.860) | 822.960(494.583-1238.833) | 704750.874(436825.062-1089053.840) | 72.960(45.223-112.745) | -0.911(-0.939--0.881) | -7.24(-7.66,-6.81) |
| Diarrheal diseases | Campylobacter | 7362715.309(3348658.652-13792456.648) | 720.708(327.787-1350.090) | 517335.856(260532.136-969584.641) | 53.558(26.972-100.377) | -0.926(-0.946--0.896) | -7.47(-7.89,-7.06) |
| Diarrheal diseases | Entamoeba | 5236789.932(2220627.488-9601520.020) | 512.609(217.369-939.856) | 423767.810(191205.898-766817.420) | 43.871(19.795-79.385) | -0.914(-0.939--0.885) | -7.25(-7.45,-7.05) |
| Diarrheal diseases | Cryptosporidium | 6589003.987(3736584.827-11531375.693) | 644.972(365.760-1128.762) | 492729.502(267120.397-819288.296) | 51.010(27.654-84.817) | -0.921(-0.942--0.895) | -6.78(-7.19,-6.37) |
| Diarrheal diseases | Rotavirus | 25858035.106(19837564.396-34082284.306) | 2531.143(1941.823-3336.183) | 1614105.070(1124262.092-2248233.149) | 167.102(116.390-232.750) | -0.934(-0.951--0.912) | -7.43(-7.97,-6.89) |
| Diarrheal diseases | Aeromonas | 2804882.830(1316322.531-5183913.462) | 274.559(128.850-507.433) | 175170.220(85489.239-318279.532) | 18.135(8.850-32.950) | -0.934(-0.955--0.910) | -7.96(-8.21,-7.70) |
| Diarrheal diseases | Clostridium difficile | 7682.798(4857.778-11176.680) | 0.752(0.476-1.094) | 9721.637(6932.979-14094.782) | 1.006(0.718-1.459) | 0.339(0.089-0.646) | 1.22(0.92,1.51) |
| Diarrheal diseases | Norovirus | 6390592.527(2612471.623-11180155.481) | 625.550(255.725-1094.382) | 501909.945(219715.909-833746.197) | 51.961(22.746-86.314) | -0.917(-0.945--0.877) | -7.45(-7.66,-7.23) |
| Diarrheal diseases | Adenovirus | 18794852.276(10429174.084-29833706.615) | 1839.755(1020.872-2920.306) | 1610937.727(977911.674-2480813.727) | 166.774(101.239-256.828) | -0.909(-0.936--0.874) | -6.87(-7.00,-6.74) |
| Lower respiratory infections | Haemophilus influenzae | 1912962.640(1596589.670-2254248.057) | 187.253(156.284-220.660) | 432463.358(357290.359-518997.790) | 44.771(36.989-53.730) | -0.761(-0.800--0.712) | -4.65(-4.88,-4.43) |
| Lower respiratory infections | Klebsiella pneumoniae | 8462493.968(7297001.893-9907167.414) | 828.361(714.275-969.774) | 1868273.365(1561100.746-2137476.052) | 193.415(161.614-221.284) | -0.767(-0.805--0.721) | -4.76(-4.95,-4.57) |
| Lower respiratory infections | Legionella spp | 13121.442(8615.763-18947.631) | 1.284(0.843-1.855) | 22960.358(16709.054-30743.784) | 2.377(1.730-3.183) | 0.850(0.607-1.220) | 1.95(1.59,2.31) |
| Lower respiratory infections | Mycoplasma | 2227870.220(1872677.205-2579349.817) | 218.078(183.309-252.483) | 492795.721(409215.168-577595.468) | 51.017(42.364-59.796) | -0.766(-0.802--0.723) | -4.74(-4.93,-4.55) |
| Lower respiratory infections | Other bacterial and viral pathogens | 1353733.042(1082240.055-1661630.821) | 132.512(105.936-162.651) | 386597.660(288838.107-476893.848) | 40.023(29.902-49.371) | -0.698(-0.755--0.642) | -4.02(-4.21,-3.84) |
| Lower respiratory infections | Pseudomonas aeruginosa | 4335522.751(3751496.559-4982052.008) | 424.388(367.220-487.674) | 1120336.794(951393.589-1286881.461) | 115.984(98.494-133.225) | -0.727(-0.772--0.681) | -4.28(-4.46,-4.09) |
| Lower respiratory infections | Staphylococcus aureus | 2469009.035(2148990.980-2833415.008) | 241.682(210.356-277.352) | 892646.865(765439.141-1007243.871) | 92.412(79.243-104.276) | -0.618(-0.681--0.554) | -3.24(-3.43,-3.05) |
| Lower respiratory infections | Streptococcus pneumoniae | 41207307.976(35320373.192-47115731.998) | 4033.624(3457.375-4611.977) | 5674640.821(4824710.057-6544160.873) | 587.472(499.482-677.490) | -0.854(-0.881--0.826) | -6.00(-6.34,-5.67) |
| Lower respiratory infections | Aspergillus spp. | 920420.722(713915.429-1179952.966) | 90.096(69.882-115.501) | 268074.931(209740.536-350333.133) | 27.753(21.714-36.269) | -0.692(-0.742--0.634) | -3.89(-4.08,-3.71) |
| Lower respiratory infections | Other Acinetobacter species | 346230.621(272879.304-424934.075) | 33.891(26.711-41.595) | 74183.287(59637.314-92243.946) | 7.680(6.174-9.550) | -0.773(-0.810--0.730) | -4.84(-5.02,-4.65) |
| Lower respiratory infections | Citrobacter spp. | 102043.765(75732.587-138317.326) | 9.989(7.413-13.539) | 45981.819(32947.075-61992.056) | 4.760(3.411-6.418) | -0.523(-0.609--0.430) | -2.63(-2.82,-2.44) |
| Lower respiratory infections | Other fungi | 602856.316(469927.464-766558.063) | 59.011(45.999-75.035) | 142073.847(107125.089-187261.302) | 14.708(11.090-19.386) | -0.751(-0.791--0.705) | -4.58(-4.79,-4.37) |
| Lower respiratory infections | Other gram-negative bacteria | 1553715.275(1342063.732-1807445.358) | 152.087(131.369-176.924) | 351270.955(297987.260-410481.052) | 36.366(30.849-42.495) | -0.761(-0.798--0.717) | -4.65(-4.84,-4.47) |
| Lower respiratory infections | Other Klebsiella species | 378387.593(318340.148-451431.549) | 37.039(31.161-44.189) | 128954.659(105848.390-149402.899) | 13.350(10.958-15.467) | -0.640(-0.698--0.576) | -3.45(-3.65,-3.26) |
| Lower respiratory infections | Morganella spp. | 77987.124(53724.250-111832.341) | 7.634(5.259-10.947) | 19198.890(13272.376-28465.378) | 1.988(1.374-2.947) | -0.740(-0.786--0.687) | -4.44(-4.62,-4.26) |
| Lower respiratory infections | Other Mycobacterium species (non-TB, non-Leprosy) | 2487179.718(1761624.341-3486063.920) | 243.460(172.439-341.237) | 756015.578(532823.414-1078077.532) | 78.267(55.161-111.609) | -0.679(-0.735--0.620) | -3.84(-4.03,-3.65) |
| Lower respiratory infections | Proteus spp. | 69852.637(56758.416-87671.422) | 6.838(5.556-8.582) | 25894.116(21297.147-32484.423) | 2.681(2.205-3.363) | -0.608(-0.664--0.534) | -3.23(-3.42,-3.04) |
| Lower respiratory infections | Serratia spp. | 177492.036(150339.530-210414.344) | 17.374(14.716-20.597) | 63779.361(51543.622-76223.208) | 6.603(5.336-7.891) | -0.620(-0.685--0.551) | -3.31(-3.53,-3.09) |
| Lower respiratory infections | Group A Streptococcus | 2027907.721(1677641.215-2379702.262) | 198.504(164.218-232.940) | 462288.716(394754.318-531291.812) | 47.859(40.867-55.002) | -0.759(-0.800--0.714) | -4.61(-4.84,-4.38) |
| Lower respiratory infections | Influenza | 3936778.962(3349314.256-4525639.547) | 385.356(327.851-442.997) | 412606.700(291624.222-595118.680) | 42.715(30.191-61.610) | -0.889(-0.924--0.831) | -5.94(-6.78,-5.10) |
| Lower respiratory infections | Respiratory syncytial virus | 4091464.436(3485415.127-4769218.796) | 400.498(341.174-466.840) | 483161.884(334432.415-707829.900) | 50.020(34.622-73.279) | -0.875(-0.917--0.804) | -5.54(-6.36,-4.72) |
| Lower respiratory infections | Group B streptococcus | 846399.529(658958.564-1094093.157) | 82.851(64.503-107.097) | 236832.321(192964.291-292746.236) | 24.518(19.977-30.307) | -0.704(-0.756--0.644) | -4.10(-4.31,-3.89) |
| Lower respiratory infections | Acinetobacter baumannii | 1622508.077(1383960.449-1915818.029) | 158.821(135.471-187.532) | 283352.465(233689.549-344845.845) | 29.334(24.193-35.700) | -0.815(-0.846--0.780) | -5.42(-5.65,-5.18) |
| Lower respiratory infections | Chlamydia spp | 1487987.068(1200947.344-1840027.435) | 145.653(117.556-180.113) | 278627.538(219143.909-337140.586) | 28.845(22.687-34.903) | -0.802(-0.835--0.762) | -5.28(-5.47,-5.09) |
| Lower respiratory infections | Enterobacter spp | 367391.136(313800.828-439250.883) | 35.962(30.717-42.997) | 122557.028(98522.278-146830.360) | 12.688(10.200-15.201) | -0.647(-0.708--0.581) | -3.53(-3.73,-3.33) |
| Lower respiratory infections | Escherichia coli | 2848025.830(2422567.657-3367300.550) | 278.782(237.136-329.612) | 890560.740(743286.430-1035734.464) | 92.196(76.949-107.225) | -0.669(-0.727--0.611) | -3.72(-3.91,-3.52) |
| Meningitis | Escherichia coli | 222464.724(165527.459-278267.181) | 21.776(16.203-27.238) | 31241.344(25113.849-38229.189) | 3.234(2.600-3.958) | -0.851(-0.885--0.803) | -5.80(-6.08,-5.52) |
| Meningitis | Haemophilus influenzae | 1255964.666(934680.189-1597284.084) | 122.942(91.492-156.352) | 55459.639(45356.067-71059.520) | 5.742(4.696-7.356) | -0.953(-0.966--0.929) | -9.01(-9.92,-8.09) |
| Meningitis | Klebsiella pneumoniae | 467737.698(346802.315-595715.580) | 45.785(33.947-58.312) | 49414.616(39680.973-64508.542) | 5.116(4.108-6.678) | -0.888(-0.916--0.847) | -6.64(-6.97,-6.31) |
| Meningitis | Group B streptococcus | 756039.143(586070.423-957302.122) | 74.006(57.368-93.707) | 107373.697(82153.956-138125.661) | 11.116(8.505-14.300) | -0.850(-0.892--0.768) | -5.64(-5.89,-5.38) |
| Meningitis | Listeria monocytogenes | 386854.143(278277.537-505502.350) | 37.868(27.240-49.482) | 48799.701(38622.724-62978.000) | 5.052(3.998-6.520) | -0.867(-0.901--0.811) | -6.09(-6.39,-5.80) |
| Meningitis | Other bacterial and viral pathogens | 396957.108(277627.628-538115.570) | 38.857(27.176-52.674) | 41632.547(32958.842-57931.252) | 4.310(3.412-5.997) | -0.889(-0.919--0.842) | -6.65(-6.98,-6.32) |
| Meningitis | Pseudomonas aeruginosa | 360047.015(263006.620-462990.030) | 35.244(25.745-45.320) | 40078.706(31752.806-52410.120) | 4.149(3.287-5.426) | -0.882(-0.913--0.834) | -6.47(-6.80,-6.15) |
| Meningitis | Staphylococcus aureus | 259105.935(204381.112-316324.461) | 25.363(20.006-30.964) | 40073.005(33317.919-49819.545) | 4.149(3.449-5.158) | -0.836(-0.871--0.781) | -5.49(-5.75,-5.23) |
| Meningitis | Streptococcus pneumoniae | 1605498.276(1207295.556-1966119.471) | 157.156(118.177-192.456) | 227274.167(186609.717-301553.797) | 23.529(19.319-31.219) | -0.850(-0.888--0.785) | -5.68(-5.95,-5.42) |
| Meningitis | Neisseria meningitidis | 1974230.521(1508272.509-2446329.993) | 193.250(147.639-239.462) | 178798.753(146252.724-230341.893) | 18.510(15.141-23.846) | -0.904(-0.928--0.868) | -7.18(-7.59,-6.77) |
| Meningitis | Other fungi | 524081.145(353478.079-759891.550) | 51.300(34.601-74.383) | 63182.074(45456.640-89675.799) | 6.541(4.706-9.284) | -0.872(-0.903--0.823) | -6.22(-6.54,-5.91) |
| Meningitis | Candida spp. | 622927.526(443609.545-820557.707) | 60.976(43.423-80.321) | 68262.668(52636.309-88222.950) | 7.067(5.449-9.133) | -0.884(-0.916--0.828) | -6.49(-6.81,-6.17) |
| Meningitis | Coagulase negative staphylococci | 554934.806(434920.751-695445.403) | 54.320(42.573-68.074) | 53180.754(42095.112-67570.291) | 5.506(4.358-6.995) | -0.899(-0.926--0.856) | -6.94(-7.27,-6.59) |
| Meningitis | Non-polio enteroviruses | 1100586.027(867945.435-1366406.331) | 107.732(84.960-133.752) | 91638.993(74950.994-115637.829) | 9.487(7.759-11.972) | -0.912(-0.932--0.879) | -7.35(-7.80,-6.91) |
| Meningitis | Other streptococcus species | 435756.082(318988.931-542751.417) | 42.654(31.225-53.128) | 55406.274(44764.235-70820.315) | 5.736(4.634-7.332) | -0.866(-0.897--0.813) | -6.06(-6.35,-5.77) |
| Meningitis | Other viral etiologies of meningitis | 700022.191(551983.747-878675.063) | 68.522(54.032-86.010) | 77073.466(64134.022-100083.771) | 7.979(6.640-10.361) | -0.884(-0.913--0.840) | -6.53(-6.85,-6.20) |
| Meningitis | Acinetobacter baumannii | 301452.347(203941.311-413981.476) | 29.508(19.963-40.523) | 32348.413(23729.560-45660.292) | 3.349(2.457-4.727) | -0.886(-0.916--0.842) | -6.59(-6.91,-6.26) |
